# Synthesis and Evaluation of the (*S*)-BINAM Derivatives as Fluorescent Enantioselective Detectors

**DOI:** 10.3390/s20113234

**Published:** 2020-06-06

**Authors:** Alexander V. Shaferov, Anna S. Malysheva, Alexei D. Averin, Olga A. Maloshitskaya, Irina P. Beletskaya

**Affiliations:** 1 Department of Chemistry, Lomonosov Moscow State University, Moscow 119991, Russia; avshaferov@gmail.com (A.V.S.); annette9513@yandex.ru (A.S.M.); maloshitskaya@mail.ru (O.A.M.); beletska@org.chem.msu.ru (I.P.B.); 2 A.N. Frumkin Institute of Physical Chemistry and Electrochemistry RAS, Moscow 119991, Russia

**Keywords:** chirality, detection, fluorescence, 1,1′-binaphthalene-2,2′-diamine, Pd catalysis, amination

## Abstract

Pd(0)-catalyzed amination was employed for the synthesis of a new family of (*S*)-1,1′-bianaphthalene-2,2′-diamine derivatives possessing additional chiral and fluorophore substituents. The Compounds thus obtained were tested as potential detectors of seven amino alcohols, and some of them were found to be able to recognize individual enantiomers of certain amino alcohols by specific changes of their emission spectra in the presence of these analytes. A pronounced dependence of the detecting abilities on the nature of the substituents in the (*S*)-BINAM derivatives was observed.

## 1. Introduction

The application of fluorescent methods for the recognition of individual enantiomers of optically active molecules has gained much attention in the recent decade and successfully competes with other spectroscopic techniques like spectrophotometric (or colorimetric) methods [1,2] or measurements of induced circular dichroism [3]. The fluorescent approach employs systematic changes in the emission spectra (quenching or enhancement, red, or blue shifts of the emission maximum) of the chiral detector as a result of molecular complex formation with a certain chiral analyte. At the same time, in the presence of enantiomer with an opposite configuration, the detector′s emission either should not change notably or change in the opposite direction in order to distinguish between two enantiomers.

Both 1,1′-binaphthalene and chiral 2,2′-binaphthol (BINOL) were shown for the first time to change their fluorescence spectra in the presence of chiral amines [4,5]. Soon after, the bis(phenylboronic) derivative of BINOL was used to detect monosaccharides [6]. Later, the fluorescence of a more complex Compound, immobilized BINOLphosphoric acid monoamide (BINOL = 2,2′-binaphthol), was studied using the same amine [7], and since then, BINOL derivatives have been developed as the most convenient fluorescent chemosensors of optically active amino acids, amino alcohols, diamines, and α-hydroxy carboxylic acids [8,9,10,11,12]. It is important that natural amino acids and their various derivatives are the most preferable analytes in almost all these studies. 

The BINOL moiety possesses inherent chirality and fluorescent properties, which make it a unique building block for creating chemosensors. Two closely positioned hydroxyl groups help to form molecular complexes with various polar organic Compounds. In order to enhance the selectivity and improve the binding properties, various modifications of BINOL have been elaborated. The introduction of substituents at positions 3 and 3′ resulted in the synthesis of chemosensors able to detect *N*-Boc-alanine and *N*-Boc-phenylalanine [13,14]. Among such substituents are amino and diamino groups [15,16,17], and *N,O*-containing linear moieties [18,19]. Additional fluorophores were also introduced in BINOL-containing molecules to take advantage of the fluorescence at longer waves like BODIPY-derived Compounds [20]. Generally speaking, there is a great variety in the structural types of fluorescent enantioselective detectors, as the applications of the macrocycles containing this moiety have been described [21,22]; and the role of synthetic and supramolecular oligomers, polymers, nanoparticles, and organometallic Compounds in the fluorescent sensing of various chiral objects has been mentioned as well [23].

Another type of enantioselective fluorescent detector is planar chiral 1,8-disubstituted naphthalenes containing two quinoline [24] or acridine [25,26,27,28] moieties. These chemosensors have been used for sensing amino acids, α-halogenosubstituted carboxylic acids, dicarboxylic acids, and 1,2-amino alcohols. In some cases, Sc(III) complexes of 1,8-(acridinyl)substituted naphthalenes were employed [29,30].

The closest nitrogen-containing analogue of BINOL, 1,1′-binapthalene-2,2′-diamine (BINAM), was shown to be efficient in the recognition of α-phenylethylamine enantiomers [31] quite long ago, but since then, it has not been developed further, unlike BINOL. Among the scarce and random applications of this molecule, one may cite the attempts to recognize tryptophan enantiomers using free BINAM [32], its combination with two residues of chiral thiourea to sense enantiomers of mandelic acid [33], and the synthesis of chiral polymers on the basis of BINOL and BINAM for the detection of phenylalanine [34]. Taking this fact into consideration, we decided to develop the synthesis of BINAM-containing detectors using palladium-catalyzed amination reactions, which have become a powerful tool for creating C(sp^2^)-N bonds in last two decades [35,36,37]. Earlier, we acquired good experience in the synthesis of macrocyclic Compounds with the help of this catalytic reaction [38,39,40], and recently, we showed the possibility of introducing endocyclic BINAM moieties in macrocyclic Compounds using this approach [41,42,43,44]. We began the investigation of the detecting abilities of the macrocyclic Compounds using individual enantiomers of 1,2-amino alcohols due to some advantages of these model Compounds: Good solubility in organic solvents, their non-ionic nature, and the presence of two different donor atoms able to form hydrogen bonds. Our preliminary investigations showed an important relationship between the structures of the BINAM-based detectors and their ability to produce a significant spectral response upon binding the analytes. In this work, we focus on the non-macrocyclic derivatives of BINAM possessing two additional chiral substituents and fluorophore moieties in order to find out the regularities in their sensing behavior.

## 2. Materials and Methods

All Pd(0)-catalyzed amination reactions were run under argon using absolute 1,4-dioxane. ^1^H and ^13^C NMR spectra were recorded on a Bruker Avance-400 spectrometer in CDCl_3_ using the residual peaks of the solvent as standards (δ 7.26 and 77.0 ppm, respectively). UV-vis spectra were obtained using an Agilent Cary 60 spectrophotometer, and the fluorescence spectra were recorded with a Hitachi F2700 spectrofluorometer. MALDI-TOF (Matrix-Assisted Laser Desorption Ionization – Time of Flight) mass spectra were obtained with a Bruker Autoflex II spectrometer using dithranol as the matrix and (polyethylene) glycols as the internal standard for precise calibration. (*S*)-1,1′-binapthalene-2,2′-diamine (BINAM) (**1**), 1,3-dibromobenzene, (*S*)-(tetrahydrofuran-2-yl)-ethanamine (**3**), *tert*-butyl (*S*)-2-(aminomethyl)pyrrolidine-1-carboxylate (**4**), (1*S*,2*S*)-2-(benzyloxy)cyclopentan-1-amine (**5**), 2-methoxyethan-1-amine (**6**), 5-(dimethylamino)naphthalene-1-sulfonyl chloride (dansyl chloride) (**11**), 4-(bromomethyl)-7-methoxy-2H-chromen-2-one (**12**), 4-(bromomethyl)-6,7-dimethoxy-2H-chromen-2-one (**22**), 6-bromoquinoline (**13**), 3-bromoquinoline (**17**), phosphine ligands Xantphos, *rac*-BINAP, DavePhos, *t*BuONa, K_2_CO_3_, and aminoalcohols as individual enantiomers were purchased from SigmaAldrich Co and used without special purification. Column chromatography was performed using silica gel 40–63 μm from Fluka Co, 1,4-dioxane was purified by distillation over sodium under argon, acetonitrile for syntheses was distilled over CaH_2_, and dichloromethane and methanol were freshly distilled prior to use. Acetonitrile of UHPLC grade was employed for UV and fluorescence experiments. Pd(dba)_2_ was synthesized using a known procedure [45]. *N,N′*-di(3-bromophenyl)-substituted (*S*)-BINAM **2** was obtained by a described method [41]. 

**Compound 7**. A two-neck flask equipped with a magnetic stirrer and reflux condenser, flushed with dry argon, was charged with Compound **2** (594 mg, 1 mmol), Pd(dba)_2_ (46 mg, 8 mol%), BINAP (56 mg, 9 mol%), and absolute dioxane (10 mL). After stirring for 2 min, amine **3** (223 mg, 2.2 mmol) and *t*BuONa (288 mg, 3 mmol) were added and the reaction mixture was refluxed for 15 h. After the reaction was over the residue was filtered and washed with dichloromethane (3 × 5 mL), combined filtrates were evaporated in vacuo, and the residue was chromatographed on silica gel using a sequence of eluents: CH_2_Cl_2_, CH_2_Cl_2_–MeOH 200:1–100:1. Target Compound **7** was obtained with CH_2_Cl_2_–MeOH 100:1 eluent. Yield 688 mg (90%), light-beige crystalline Compound, m. p. 92–95 °C. ^1^H-NMR (400 MHz, CDCl_3_): δ 1.60 (dq, 2H, *^2^J* = 12.2 Hz, *^3^J* = 7.3 Hz, CH_2_), 1.85–2.04 (m, 6H, CH_2_), 2.99 (dd, 2H, *^2^J* = 12.4 Hz, *^3^J* = 8.2 Hz, CH_2_N), 3.14 (dd, 2H, *^2^J* = 12.4 Hz, *^3^J* = 3.7 Hz, CH_2_N), 3.77 (td, 2H, *^2^J* = 7.5 Hz, *^3^J* = 7.5 Hz, *^3^J* = 7.0 Hz, CH_2_O), 3.87 (q, 2H, *J_obs_* = 7.3 Hz, CH_2_O), 4.08 (qd, 2H, *^3^J* = 7.0 Hz, *^3^J* = 3.7 Hz, CHO), 5.53 (s, 2H, NH), 6.24–6.33 (br. m, 4H, H(Ph)), 6.37 (d, 2H, *^3^J* = 8.2 Hz, H(Ph)), 6.95 (t, 2H, *^3^J* = 8.2 Hz, H5, H5′(Ph)), 7.11 (d, 2H, *^3^J* = 8.2 Hz, H3, H3′(BiNp)), 7.20–7.25 (m, 2H, H(BiNp)), 7.29–7.34 (m, 2H, H(BiNp)), 7.70 (d, 2H, *^3^J* = 9.0 Hz, H8, H8′(BiNp)), 7.84 (d, 2H, *^3^J* = 8.2 Hz, H4, H4′(BiNp)), 7.87 (d, 2H, *^3^J* = 9.0 Hz, H5, H5′(BiNp)), two NH protons were not assigned. ^13^C-NMR (100.6 MHz, CDCl_3_): δ 25.7 (2CH_2_), 29.0 (2CH_2_), 48.1 (2CH_2_N), 68.0 (2CH_2_O), 77.4 (2CHO), 104.5 (2CH(Ph)), 107.5 (2CH(Ph)), 109.4 (2CH(Ph)), 116.2 (2C(BiNp)), 118.3 (2CH(BiNp)), 123.2 (2CH(BiNp)), 124.5 (2CH(BiNp)), 126.8 (2CH(BiNp)), 128.1 (2CH(BiNp)), 129.1 (2CH(Ph)), 129.2 (2C(BiNp)), 129.8 (2C(BiNp)), 133.9 (2C(BiNp)), 140.5 (2NC(Ar)), 143.5 (2NC(Ar)), 149.2 (2NC(Ar)). MALDI-TOF calcd for C_42_H_43_N_4_O_2_ [M + H]^+^ 635.3386, found 635.3362.

**Compound 8**. A two-neck flask equipped with a magnetic stirrer and reflux condenser, flushed with dry argon, was charged with Compound **2** (594 mg, 1 mmol), Pd(dba)_2_ (46 mg, 8 mol%), BINAP (56 mg, 9 mol%), and absolute dioxane (10 mL). After stirring for 2 min, amine **4** (450 mg, 2.2 mmol) and *t*BuONa (288 mg, 3 mmol) were added and the reaction mixture was refluxed for 15 h. After the reaction was over, the residue was filtered, washed with dichloromethane (3 × 5 mL), combined filtrates were evaporated in vacuo, and the residue was chromatographed on silica gel using a sequence of eluents: CH_2_Cl_2_, CH_2_Cl_2_–MeOH 200:1–50:1. Target Compound **8** was obtained with CH_2_Cl_2_–MeOH 100:1 eluent. Yield 706 mg (85%), light-beige crystalline Compound, m. p. 114–116 °C. ^1^H-NMR (400 MHz, CDCl_3_, mixture of rotamers): δ 1.46 (br. s, 18H, CH_3_), 1.65–1.75 (br. m, 2H, CH_2_), 1.76–1.90 (br. m, 4H, CH_2_), 1.90–2.01 (br. m, 2H, CH_2_), 2.91 br. s + (2.97–3.12) br. m + 3.23 br. s + 3.33 br. s + 3.45 br. s (8H, CH_2_N), 3.99 br. s + 4.14 br. s (2H, CHN), 5.55 (s, 2H, NH), 6.19 (br. s, 2H, H(Ph)), 6.27 (br. d, 2H, *^3^J_obs_* = 7.2 Hz, H(Ph)), 6.36 (br. s, 2H, H(Ph)), 6.99 (t, 2H, *^3^J* = 8.0 Hz, H5, H5′(Ph)), 7.14 (d, 2H, *^3^J* = 8.3 Hz, H3, H3′(BiNp)), 7.13–7.18 (m, 2H, H(BiNp)), 7.27–7.32 (m, 2H, H(BiNp)), 7.73 (d, 2H, *^3^J* = 8.7 Hz, H8, H8′(BiNp)), 7.82–7.87 (m, 4H, H4, H4′, H5, H5′(BiNp)), two NH protons were not assigned. ^13^C-NMR (100.6 MHz, CDCl_3_, mixture of rotamers): δ 22.8 + 23.9 (2CH_2_), 28.4 (6CH_3_), 29.3 (2CH_2_), 46.5 + 46.9 + 49.4 br. (4CH_2_N), 55.0 + 56.4 (2CHN), 79.6 + 79.9 (2C(*t*-Bu)), 104.1 br. (2CH(Ph)), 106.7 br. + 107.1 br. (2CH(Ph)), 109.0 br. (2CH(Ph)), 116.1 br. (2C(Nf)), 118.3 (2CH(Nf)), 123.1 (2CH(Nf)), 124.5 (2CH(Nf)), 126.8 (2CH(Nf)), 128.1 (2CH(Nf)), 129.1 (2CH(Ph)), 129.2 (2C(Nf)), 129.7 (2CH(Nf)), 134.0 (2C(Nf)), 140.6 br. (2NC(Ar)), 143.5 br. (2NC(Ar)), 149.1 br. (2NC(Ar)), 154.7 br. + 156.0 (2CO). MALDI-TOF calcd for C_52_H_61_N_6_O_4_ [M + H]^+^ 833.4754, found 833.4817.

**Compound 9**. A two-neck flask equipped with a magnetic stirrer and reflux condenser, flushed with dry argon, was charged with Compound **2** (119 mg, 0.2 mmol), Pd(dba)_2_ (18 mg, 16 mol%), BINAP (22 mg, 18 mol%), and absolute dioxane (2 mL). After stirring for 2 min, amine **5** (84 mg, 0.44 mmol) and *t*BuONa (58 mg, 0.6 mmol) were added and the reaction mixture was refluxed for 15 h. After the reaction was over, the residue was filtered, washed with dichloromethane (3 × 3 mL), combined filtrates were evaporated in vacuo, and the residue was chromatographed on silica gel using a sequence of eluents: CH_2_Cl_2_, CH_2_Cl_2_–MeOH 200:1–5:1. Target Compound **9** was obtained with CH_2_Cl_2_ eluent. Yield 129 mg (79%), yellow glassy Compound. ^1^H-NMR (400 MHz, CDCl_3_): δ 1.14 (br. s, 2H, CH_2_), 1.65–1.88 (m, 8H, CH_2_), 2.21 (sextet, 2H, *^3^J* = 7.0 Hz, CH_2_), 3.48 (br. s, 2H, NH), 3.67–3.74 (m, 2H, CH_2_N), 3.75–3.80 (m, 2H, CHO), 4.44 (d, 2H, *^2^J* = 11.7 Hz, CH_2_O), 4.51 (d, 2H, *^2^J* = 11.7 Hz, CH_2_O), 5.57 (s, 2H, NH), 6.23–6.28 (m, 4H, H(Ph)), 6.34 (d, 2H, *^3^J* = 8.6 Hz, H(Ph)), 7.19 (t, 2H, *^3^J* = 7.9 Hz, H(Ph)), 7.16–7.21 (m, 4H, H(Ph), H(BiNp)), 7.22–7.28 (m, 10H, H(Ph), H(BiNp)), 7.29–7.35 (m, 2H, H(BiNp)), 7.76 (d, 2H, *^3^J* = 8.9 Hz, H(BiNp)), 7.86 (d, 2H, *^3^J* = 8.1 Hz, H(BiNp)), 7.88 (d, 2H, *^3^J* = 8.9 Hz, H(BiNp)). ^13^C-NMR (100.6 MHz, CDCl_3_): δ 21.8 (2CH_2_), 30.0 (2CH_2_), 31.7 (2CH_2_), 59.9 (2CHN), 71.0 (2CH_2_O), 85.0 (2CHO), 104.7 (2CH(Ph)), 107.3 (2CH(Ph)), 109.3 (2CH(Ph)), 118.4 (2CH(BiNp)), 119.9 (2C(BiNp)), 123.4 (2CH(BiNp)), 124.5 (2CH(BiNp)), 126.9 (2CH(Ph)), 127.5 (2CH(BiNp)), 127.6 (4CH(Ph)), 128.1 (2CH(BiNp)), 128.3 (4CH(Ph)), 129.2 (2CH(Ph)), 129.3 (2C(BiNp)), 129.8 (2CH(BiNp)), 134.0 (2C(BiNp)), 138.4 (2C(Ph)), 140.5 (2NC(Ar)), 143.5 (2NC(Ar)), 148.6 (2NC(Ar)). MALDI-TOF calcd for C_56_H_55_N_4_O_2_ [M + H]^+^ 815.4325, found: 815.4369.

**Compound 10**. A two-neck flask equipped with a magnetic stirrer and reflux condenser, flushed with dry argon, was charged with Compound **2** (594 mg, 1 mmol), Pd(dba)_2_ (46 mg, 8 mol%), BINAP (56 mg, 9 mol%), and absolute dioxane (10 mL). After stirring for 2 min, amine **6** (165 mg, 2.2 mmol) and *t*BuONa (288 mg, 3 mmol) were added and the reaction mixture was refluxed for 15 h. After the reaction was over, the residue was filtered, washed with dichloromethane (3 × 5 mL), combined filtrates were evaporated in vacuo, and the residue was chromatographed on silica gel using a sequence of eluents: CH_2_Cl_2_, CH_2_Cl_2_–MeOH 200:1–50:1. Target Compound **10** was obtained with CH_2_Cl_2_–MeOH 100:1 eluent. Yield 688 mg (89%), light-beige crystalline Compound, m. p. 66–68 °C. ^1^H-NMR (400 MHz, CDCl_3_): δ 3.20 (t, 4H, *^3^J* = 5.2 Hz, CH_2_N), 3.39 (s, 6H, CH_3_), 3.57 (t, 4H, *^3^J* = 5.2 Hz, CH_2_O), 3.93 (br. s, 2H, NH), 5.63 (s, 2H, NH), 6.24–6.29 (m, 4H, H(Ph)), 6.43 (dd, 2H, *^3^J* = 7.9 Hz, *^4^J* = 1.0 Hz, H(Ph)), 7.04 (t, 2H, *^3^J* = 7.9 Hz, H5, H5′(Ph)), 7.20 (d, 2H, *^3^J* = 8.4 Hz, H3, H3′(BiNp)), 7.19–7.23 (m, 2H, H(BiNp)), 7.32–7.36 (m, 2H, H(BiNp)), 7.78 (d, 2H, *^3^J* = 9.0 Hz, H8, H8′(BiNp)), 7.89 (d, 2H, *^3^J* = 8.4 Hz, H4, H4′(BiNp)), 7.91 (d, 2H, *^3^J* = 9.1 Hz, H5, H5′(BiNp)). ^13^C-NMR (100.6 MHz, CDCl_3_): δ 43.3 (2CH_2_N), 58.6 (2CH_3_), 70.8 (2CH_2_O), 104.4 (2CH(Ph)), 107.3 (2CH(Ph)), 109.4 (2CH(Ph)), 116.2 (2CH(BiNp)), 118.3 (2CH(BiNp)), 123.2 (2CH(BiNp)), 124.4 (2CH(BiNp)), 126.8 (2CH(BiNp)), 128.0 (2CH(BiNp)), 129.1 (2CH(Ph)), 129.2 (2C(BiNp)), 129.8 (2CH(BiNp)), 133.9 (2C(BiNp)), 140.4 (2NC(Ar)), 143.5 (2NC(Ar)), 149.1 (2NC(Ar)). MALDI-TOF calcd for C_38_H_39_N_4_O_2_ [M + H]^+^ 583.3073, found: 583.3033.

**Compound 14**. A one-neck flask equipped with a magnetic stirrer was charged with Compound **7** (127 mg, 0.2 mmol), acetonitrile (2 mL), dansyl chloride **11** (108 mg, 0.4 mmol), K_2_CO_3_ (110 mg, 0.8 mmol), and the reaction mixture was stirred for 8 h at room temperature. The residue was filtered, washed with dichloromethane (3 × 5 ml), and the combined organic fractions were evaporated in vacuo. The target Compound **14** was obtained as a yellow crystalline powder. Yield 219 mg (98%), m.p. 140–142°C (decomp.). ^1^H-NMR (400 MHz, CDCl_3_): δ 1.61–1.70 (m, 2H, CH_2_), 1.75–1.92 (m, 6H, CH_2_), 2.88 (12H, CH_3_), 3.58–3.70 (m, 6H, CH_2_O, CH_2_N), 3.70–3.76 (m, 2H, CH_2_O), 3.83–3.89 (m, 2H, CHO), 5.44 (s, 2H, NH), 6.70 (d, 2H, *^3^J* = 7.5 Hz, H(Ph)), 6.72 (d, 2H, *^3^J* = 7.7 Hz, H(Ph)), 6.80 (br. s, 2H, H2(Ph)), 7.01 (t, 2H, *^3^J* = 7.5 Hz, H5, H5′(Ph)), 7.04 (d, 2H, *^3^J* = 8.5 Hz, H3, H3′(biNp)), 7.12 (d, 2H, *^3^J* = 7.3 Hz, H6, H6′(Dans)), 7.15–7.22 (m, 4H, H(biNp)), 7.29–7.37 (m, 4H, H3, H3′, H7, H7′(Dans)), 7.41 (t, 2H, *^3^J_obs_* = 7.8 Hz, H(biNP)), 7.77 (d, 2H, *^3^J* = 8.9 Hz, H(biNP)), 7.85 (d, 2H, *^3^J* = 7.8 Hz, H(biNP)), 8.08–8.13 (m, 4H, H2, H2′, H8, H8′(Dans)), 8.54 (d, 2H, *^3^J* = 8.5 Hz, H4, H4′(Dans)). ^13^C-NMR (100.6 MHz, CDCl_3_): δ 25.3 (2CH_2_), 29.1 (2CH_2_), 45.3 (4CH_3_), 54.0 (2CH_2_N), 67.9 (2CH_2_O), 76.4 (2CHO), 115.0 (2CH(Ar)), 116.4 (2C(Np)), 117.3 (2CH(Ar)), 119.0 (2CH(Ar)), 119.6 (2CH(Ar)), 119.9 (2CH(Ar)), 122.5 (2CH(Ar)), 123.0 (2CH(Ar)), 123.6 (2CH(Ar)), 124.2 (2CH(Ar)), 127.0 (2CH(Ar)), 127.7 (2CH(Ar)), 128.1 (2CH(Ar)), 129.3 (2C(Np)), 129.4 (2CH(Ar)), 129.5 (2CH(Ar)), 129.7 (2C(Np)), 130.1 (2C(Np)), 130.3 (2CH(Ar)), 130.7 (2CH(Ar)), 133.5 (2C(Np)), 134.1 (2C(Np)), 139.4 (2NC(Ar)), 140.0 (2NC(Ar)), 142.8 (2NC(Ar)), 151.3 (C5, C5′(Dans)). MALDI-TOF calcd for C_66_H_65_N_6_O_6_S_2_ [M + H]^+^ 1101.4407, found: 1101.4346.

**Compound 15**. A one-neck flask equipped with a magnetic stirrer was charged with Compound **7** (95 mg, 0.15 mmol), acetonitrile (1 mL), 4-bromo-7-methoxycoumarin **12** (82 mg, 0.3 mmol), and K_2_CO_3_ (103 mg, 0.75 mmol), and the reaction mixture was stirred for 8 h at 50 °C. The residue was filtered, washed with dichloromethane (3 × 5 mL), and the combined organic fractions were evaporated in vacuo. The target Compound **15** was obtained as a brownish viscous oil. Yield 149 mg (98%). ^1^H-NMR (400 MHz, CDCl_3_): δ 1.48 (dq, 2H, *^2^J* = 11.9 Hz, *^3^J* = 8.2 Hz, CH_2_), 1.78–1.89 (m, 6H, CH_2_), 1.92–2.00 (m, 2H, CH_2_), 3.25 (dd, 2H, *^2^J* = 15.4 Hz, *^3^J* = 7.6 Hz, CH_2_N), 3.56 (dd, 2H, *^2^J* = 15.4 Hz, *^3^J* = 2.8 Hz, CH_2_N), 3.66–3.72 (m, 2H, CH_2_O), 3.77–3.83 (m, 2H, CH_2_O), 3.82 (s, 6H, CH_3_O), 4.13 (qd, 2H, *^3^J* = 7.3 Hz, *^3^J* = 2.8 Hz, CHO), 4.65 (br. s, 4H, CoumCH_2_N), 5.48 (br. s, 2H, NH), 6.07 (s, 2H, HC=), 6.14 (br. s, 2H, H2, H2′(Ph)), 6.20 (d, 2H, *^3^J* = 8.2 Hz, H(Ph)), 6.32 (d, 2H, *^3^J* = 7.9 Hz, H(Ph)), 6.77–6.81 (m, 4H, H6, H6′, H8, H8′(Coum)), 6.96 (t, 2H, *^3^J* = 8.1 Hz, H5, H5′(Ph)), 7.01 (d, 2H, *^3^J* = 8.4 Hz, H3, H3′(BiNp)), 7.12–7.16 (m, 2H, H6, H6′(BiNp)), 7.21–7.25 (m, 2H, H7, H7′(BiNp)), 7.43 (d, 2H, *^3^J* = 8.6 Hz, H5, H5′(Coum)), 7.57 (d, 2H, H8, H8′(BiNp)), 7.73 (d, 2H, *^3^J* = 9.1 Hz, H5, H5′(BiNp)), 7.75 (d, 2H, *^3^J* = 8.4 Hz, H4, H4′(BiNp)). ^13^C-NMR (100.6 MHz, CDCl_3_): δ 25.4 (2CH_2_), 29.1 (2CH_2_), 51.3 (2CH_2_N), 55.0 (2CoumCH_2_N), 55.6 (2CH_3_O), 67.9 (2CH_2_O), 77.9 (2CHO), 104.0 (2CH(Ar)), 106.3 (2CH(Ar)), 109.0 (2CH(Ar)), 109.2 (2CH(Ar)), 110.9 (C3, C3′(Coum)), 111.4 (C4a, C4a′(Coum)), 116.5 (2C(BiNp)), 118.5 (2C3, C3′(Ar)), 120.1 (2CH(Ar)), 123.1 (2CH(Ar)), 124.1 (2CH(Ar)), 124.2 (2CH(Ar)), 126.6 (2CH(Ar)), 128.0 (2CH(Ar)), 128.9 (2CH(Ar)), 129.1 (2C(BiNp)), 129.8 (2CH(Ar)), 133.7 (2C(Ar)), 140.4 (2C(Ar)), 143.7 (2NC(Ar)), 148.5 (2NC(Ar)), 151.6 (2NC(Ar)), 155.4 (2CO), 161.2 (2CO), 162.4 (2CO). MALDI-TOF calcd for C_64_H_59_N_4_O_8_ [M + H]^+^ 1011.4333, found: 1011.4297.

**Compound 16.** A two-neck flask equipped with a magnetic stirrer and reflux condenser, flushed with dry argon, was charged with Compound **7** (95 mg, 0.15 mmol), Pd(dba)_2_ (14 mg, 16 mol%), DavePhos (11 mg, 18 mol%), and absolute dioxane (2 mL). After stirring for 2 min, 6-bromoquinoline **12** (125 mg, 0.6 mmol) and *t*BuONa (72 mg, 0.75 mmol) were added and the reaction mixture was refluxed for 15 h. After the reaction was over the residue was filtered, washed with dichloromethane (3 × 5 mL), the combined filtrates were evaporated in vacuo, and the residue was chromatographed on silica gel using a sequence of eluents: CH_2_Cl_2_, CH_2_Cl_2_–MeOH 200:1–100:1. Target Compound **16** was obtained with CH_2_Cl_2_–MeOH 100:1 eluent. Yield 77 mg (58%), beige crystalline Compound, m. p. 121–124 °C (decomp.). ^1^H-NMR (400 MHz, CDCl_3_): δ 1.50 (dq, 2H, *^2^J* = 12.0 Hz, *^3^J* = 7.7 Hz, CH_2_), 1.77–1.98 (m, 6H, CH_2_), 3.71 (td, 2H, *^3^J* = 7.8 Hz, *^3^J* = 6.3 Hz, CH_2_O), 3.79–3.87 (m, 6H, CH_2_N, CH_2_O), 4.17 (quin, 2H, *^3^J_obs_* = 6.4 Hz, CHO), 5.59 (s, 2H, NH), 6.65 (dd, 2H, *^3^J* = 7.9 Hz, *^4^J* = 1.9 Hz, H(Ph)), 6.73 (dd, 2H, *^3^J* = 7.9 Hz, *^4^J* = 1.9 Hz, H(Ph)), 6.78 (t, 2H, *^4^J* = 1.9 Hz, H2, H2′(Ph)), 7.08 (d, 2H, *^3^J* = 8.1 Hz, H3, H3′(biBiNp)), 7.09 (t, 2H, *^3^J* = 7.9 Hz, H5, H5′(Ph)), 7.18 (ddd, 2H, *^3^J* = 9.1 Hz, *^3^J* = 6.8 Hz, *^4^J* = 1.2 Hz, H6, H6′(BiNp)), 7.23 (d, 2H, *^4^J* = 2.6 Hz, H5, H5′(Quin)), 7.25–7.31 (m, 4H, H7, H7′(BiNp), H3, H3′(Quin)), 7.40 (dd, 2H, *^3^J* = 9.3 Hz, *^4^J* = 2.6 Hz, H7, H7′(Quin)), 7.70 (d, 2H, *^3^J* = 9.0 Hz, H8, H8′(BiNp)), 7.81 (d, 2H, *^3^J* = 8.1 Hz, H4, H4′(BiNp)), 7.85 (d, 2H, *^3^J* = 9.1 Hz, H5, H5′(BiNp)), 7.91 (d, 2H, *^3^J* = 9.3 Hz, H8, H8′(Quin)), 7.95 (d, 2H, *^3^J* = 8.4 Hz, H4, H4′(Quin)), 8.69 (dd, 2H, *^3^J* = 3.8 Hz, *^4^J* = 0.8 Hz, H2, H2′(Quin)). ^13^C-NMR (100.6 MHz, CDCl_3_): δ 25.6 (2CH_2_), 29.7 (2CH_2_), 56.8 (2CH_2_N), 67.9 (2CH_2_O), 76.6 (2CHO), 112.6 (2CH(Ar)), 114.2 (2CH(Ar)), 114.3 (2CH(Ar)), 116.7 (2C(Ar)), 116.8 (2CH(Ar)), 118.0 (2CH(Ar)), 121.2 (2CH(Ar)), 123.5 (2CH(Ar)), 124.4 (2CH(Ar)), 125.0 (2CH(Ar)), 127.0 (2CH(Ar)), 128.1 (2CH(Ar)), 129.3 (2CH(Ar)), 129.4 (4C(Ar)), 129.5 (2CH(Ar)), 130.1 (2CH(Ar)), 133.8 (2C(Ar)), 134.8 (2CH(Ar)), 139.8 (2C(Ar)), 143.7 (2C(Ar)), 143.8 (2C(Ar)), 146.2 (2C(Ar)), 147.2 (2CH(Ar)), 148.6 (2C(Ar)). MALDI-TOF calcd for C_60_H_53_N_6_O_2_ [M + H]^+^ 889.4230, found: 889.4157.

**Compound 17**. A one-neck flask equipped with a magnetic stirrer was charged with Compound **8** (165 mg, 0.2 mmol), acetonitrile (2 mL), dansyl chloride **11** (108 mg, 0.4 mmol), and K_2_CO_3_ (110 mg, 0.8 mmol), and the reaction mixture was stirred for 8 h at room temperature. The residue was filtered, washed with dichloromethane (3 × 5 mL), and the combined organic fractions were evaporated in vacuo. The target Compound **17** was obtained as a yellow crystalline powder. Yield 257 mg (99%), m.p. 152–154 °C (decomp.). ^1^H-NMR (400 MHz, CDCl_3_, mixture of rotamers): δ 1.10 + 1.32 (s, 18H, CH_3_C), 1.70–1.83 (br. m, 6H), 2.07 + 2.16 (br. s, 2H, CH_2_), 2.86 (s, 12H, CH_3_N), (3.20–3.25) m + 3.30 br. s (4H, CH_2_N), (3.50–3.58) m + 3.63 br. s + (3.65–3.79) (6H, CH_2_N, CHN), 5.44 + 5.46 (br. s, 2H, NH), (6.61–6.75) m + 6.80 d (*^3^J* = 7.8 Hz) + 6.92 br. s (6H, H(Ph)), 6.95–7.12 (m, 6H, H(Np)), 7.15–7.23 (m, 4H, H(Np)), 7.27–7.34 (m, 4H, H(Np)), 7.35–7.43 (m, 2H, H(Np)), 7.80–7.87 (m, 4H, H(Np)), 7.98 (d, *^3^J* = 8.4 Hz) + 8.05 (d, *^3^J_obs_* = 7.3 Hz) (4H, H2, H2′, H8, H8′(Dans)), 8.51 (d, *^3^J* = 8.7 Hz) + 8.53 (d, *^3^J* = 8.5 Hz) (2H, H4, H4′(Dans)). ^13^C-NMR (100.6 MHz, CDCl_3_, mixture of rotamers): δ 22.3 + 23.3 (2CH_2_), 27.4 + 28.1 +28.3 (2CH_2_ + 6CH_3_(*t*-Bu)), 45.3 (4CH_3_N), 46.3 + 46.9 (2CH_2_N), 50.2 + 51.3 (2CH_2_N), 55.2 (2CHN), 78.9 + 79.2 (2C(*t*-Bu)), 115.0 (2CH(Ar)), 116.1 (2C(biNp)), 117.0 br. + 117.4 br. (2CH(Ar)), 119.0 br. + 119.2 br. + 119.6 + 119.8 + 120.3 br. (6CH(Ar)), 121.8 br. + 122.3 br. (2CH(Ar)), 123.0 (2CH(Ar)), 123.5 br. + 123.6 br. (2CH(Ar)), 124.2 (2CH(Ar)), 127.1 br. (2CH(Ar)), 127.6 + 127.7 (2CH(Ar)), 128.1 (2CH(Ar)), 129.4 br. (4CH(Ar)), 129.7 (2C(Nf)), 130.1 (2C(Nf)), 130.3 + 130.4 (2CH(Ar)), 130.6 (2C(Nf)), 130.8 (2CH(Ar)), 133.5 (2C(Nf)), 133.9 + 134.1 (2C(Nf)), 139.3 + 139.4 + 139.5 + 139.7 (4NC(Ar)), 142.8 br. (2NC(Ar)), 151.3 + 151.4 (C5, C5′(Dans)), 154.0 + 154.2 (2CO). MALDI-TOF calcd for C_76_H_83_N_8_O_8_S_2_ [M + H]^+^ 1299.5775, found: 1299.5712.

**Compound 18**. A one-neck flask equipped with a magnetic stirrer was charged with Compound **8** (125 mg, 0.15 mmol), acetonitrile (1 mL), 4-bromo-7-methoxycoumarin **12** (82 mg, 0.3 mmol), and K_2_CO_3_ (103 mg, 0.75 mmol), and the reaction mixture was stirred for 8 h at 50 °C. The residue was filtered, washed with dichloromethane (3 × 5 mL), combined organic fractions were evaporated in vacuo, and the residue was chromatographed on silica gel using a sequence of eluents: CH_2_Cl_2_, CH_2_Cl_2_–MeOH 200:1–50:1. Target Compound **18** was obtained with CH_2_Cl_2_–MeOH 100:1 eluent. Yield 130 mg (71%), yellow glassy Compound. ^1^H-NMR (400 MHz, CDCl_3_, mixture of rotamers): δ 1.28 (s, 9H, CH_3_), 1.36 (s, 9H, CH_3_), 1.75–1.97 (br. m, 8H, CH_2_), 3.05–3.20 (m, 2H, CH_2_N), 3.27 (br. s, 2H, CH_2_N), 3.38 (br. s, 2H, CH_2_N), 3.48–3.60 (m, 2H, CH_2_N), 3.83 (s, 6H, CH_3_O), 4.07 + 4. 14 (br. s, 2H, CHN), 4.43 + 4.48 (br. s, 4H, PhCH_2_N), 5.34 + 5.50 + 5.58 (br. s, 2H, NH), 6.00 + 6.04 (s, 2H, =CH(Coum)), 6.24–6.41 (m, 4H, H6, H6′, H8, H8′(Coum)), 6.73–6.84 (m, 6H, H(Ph)), 6.95–7.04 (m, 4H, H5, H5′(Ph), H3, H3′(BiNp)), 7.06–7.25 (m, 4H, H6, H6′, H7, H7′(BiNp)), 7.31–7.50 (m, 4H, H(BiNp), H5, H5′(Coum)), 7.63–7.69 (m, 2H, H(BiNp)), 7.73 (d, 2H, *^3^J* = 8.0 Hz, H(BiNp)). ^13^C-NMR (100.6 MHz, CDCl_3_, mixture of rotamers): δ 22.6 + 23.5 (2CH_2_), 28.3 (6CH_3_), 28.6 + 28.9 (2CH_2_), 46.2 + 46.7 (2CH_2_N), 51.6 + 53.3 + 53.8 + 55.3 (2PhCH_2_N, 2CHN), 55.6 (2CH_3_O), 79.1 + 79.9 (2C(*t-*Bu)), 100.8 + 101.1 (C3, C3′(Coum)), 104.0–104.5 м. (4C), 106.4 (2C), 109.4 (2C), 111.1 + 111.3 (2C), 112.0 + 112.2 (2C), 117.1 (2C), 118.7 + 119.0 (2C), 123.2 (2C), 133.8 + 124.1 + 124.3 (4C), 126.6 (2C), 128.0 (2C), 128.8 + 128.9 + 129.0 (4C), 129.3 (2C), 129.8 (2C), 133.8 (2C), 140.4 (2C), 144.0 + 144.2 (2C), 148.1 + 148.5 (2C), 150.9 + 151.1 (2C), 151.5 + 151.6 (2C), 154.4 + 155.4 (2CO(*t-*Bu)), 161.1 + 162.5 (C2, C2′(Coum)). MALDI-TOF calcd for C_74_H_77_N_6_O_10_ [M + H]^+^ 1209.5701, found: 1209.5765.

**Compound 19**. A two-neck flask equipped with a magnetic stirrer and reflux condenser, flushed with dry argon, was charged with Compound **8** (125 mg, 0.15 mmol), Pd(dba)_2_ (14 mg, 16 mol%), DavePhos (11 mg, 18 mol%), and absolute dioxane (2 mL). After stirring for 2 min, 6-bromoquinoline **12** (125 mg, 0.6 mmol) and *t*BuONa (72 mg, 0.75 mmol) were added and the reaction mixture was refluxed for 15 h. After the reaction was over, the residue was filtered, washed with dichloromethane (3 × 5 mL), combined filtrates were evaporated in vacuo, and the residue was chromatographed on silica gel using a sequence of eluents: CH_2_Cl_2_, CH_2_Cl_2_–MeOH 200:1–100:1. Target Compound **19** was obtained with CH_2_Cl_2_–MeOH 100:1 eluent. Yield 68 mg (41%), yellow viscous oil. ^1^H-NMR (400 MHz, CDCl_3_, mixture of rotamers): δ 1.38 (s, 9H, CH_3_), 1.45 (s, 9H, CH_3_), 1.66–1.95 (br. m, 8H, CH_2_), 3.25 br. s + 3.32–3.40 br. m (4H, CH_2_N), 3.40–3.46 br. m + 3.52 br. s (2H, CHN), 4.02 d (*^2^J* = 12.4 Hz) + 4.13 br. d (*^2^J* = 12.4 Hz) (2H, CH_2_N), 4.21 (br. s, 2H, CH_2_N), 5.53 + 5.65 + 5.67 (s, 2H, NH), 6.60–6.65 m + 6.71 d (*^3^J* = 8.0 Hz) (4H, H(Ph)), 6.67 + 6.85 (br. s, 2H, H2, H2′(Ph)), 7.05–7.19 m +7.54 br. s (8H, H5, H5′(Ph), H5, H5′(Quin), H3, H3′, H6, H6′(Np)), 7.24–7.28 m + 7.28–7.33 br. m 7.36–7.41 br. m (4H, H7, H7′(Np), H3, H3′(Quin)), 7.62–7.70 br. m + 7.80 d (*^3^J* = 8.3 Hz), 7.80–7.85 br. m (6H, H4, H4′, H5, H5′, H8, H8′(Np)), 7.88–7.98 br. m + 8.06 br. d (*^3^J_obs_* = 8.0 Hz) (4H, H8, H8′, H4, H4′(Quin)), 8.69 (br. s, 2H, H2, H2′(Quin)). ^13^C-NMR (100.6 MHz, CDCl_3_, mixture of rotamers): δ 22.6 + 23.5 (2CH_2_), 28.4 + 28.6 (6CH_3,_ 2CH_2_), 46.4 + 46.7 (2CH_2_N), 54.3 + 54.5 (2CH_2_N), 55.3 (2CHN), 79.1 + 79.9 (2C(*t-*Bu)), 112.6 + 113.0 (2CH(Ar)), 114.1 + 114.4 + 114.8 (4CH(Ar)), 116.4 + 116.8 (2C(Ar), 2CH(Ar)), 118.0 + 118.3 (2CH(Ar)), 121.0 + 121.4 (2CH(Ar)), 123.6 (2CH(Ar)), 124.5 + 125.3 (4CH(Ar)), 126.9 + 127.0 (2CH(Ar)), 128.2 (2CH(Ar)), 129.4 + 129.8 (4CH(Ar), 4C(Ar)), 130.3 (2CH(Ar)), 133.8 + 134.7 (2CH(Ar)), 135.9 + 136.1 (2C(Ar)), 139.8 (2C(Ar)), 144.0 (4C(Ar)), 146.5 + 147.6 (2CH(Ar), 2C(Ar)), 149.0 (2C(Ar)), 154.4 + 154.7 (2CO). MALDI-TOF calcd for C_70_H_71_N_8_O_4_ [M + H]^+^ 1087.5598, found: 1087.5663.

**Compound 22**. A one-neck flask equipped with a magnetic stirrer was charged with Compound **10** (87 mg, 0.15 mmol), acetonitrile (1 mL), dansyl chloride **11** (82 mg, 0.3 mmol), and K_2_CO_3_ (13 mg, 0.75 mmol), and the reaction mixture was stirred for 8 h at room temperature. The residue was filtered, washed with dichloromethane (3 × 5 mL) and the combined organic fractions were evaporated in vacuo. The target Compound **22** was obtained as a brownish crystalline powder. Yield 130 mg (83%), m.p. 120–122 °C. ^1^H-NMR (400 MHz, CDCl_3_): δ 2.89 (s, 12H, CH_3_N), 3.19 (s, 6H, CH_2_O), 3.38 (t, 4H, *^3^J* = 6.3 Hz, CH_2_N), 3.75 (t, 4H, *^3^J* = 6.3 Hz, CH_2_O), 5.45 (br. s, 2H, NH), 6.87 (dd, 2H, *^3^J* = 8.0 Hz, *^4^J* = 2.0 Hz, H(Ph)), 6.71 (dd, 2H, *^3^J* = 8.0 Hz, *^4^J* = 2.0 Hz, H(Ph)), 6.76 (t, 2H, *^4^J* = 2.0 Hz, H2, H2′(Ph)), 7.01 (t, 2H, *^3^J* = 8.0 Hz, H5, H5′(Ph)), 7.02 (d, 2H, *^3^J* = 8.5 Hz, H(Np)), 7.13 (d, 2H, *^3^J* = 7.5 Hz, H(Np)), 7.19 (ddd, 2H, *^3^J* = 8.2 Hz, *^3^J* = 6.9 Hz, *^4^J* = 1.2 Hz, H(Np)), 7.20 (d, 2H, *^3^J* = 8.9 Hz, H(Np)), 7.32 (ddd, 2H, *^3^J* = 8.0 Hz, *^3^J* = 6.9 Hz, *^4^J* = 1.0 Hz, H(Np)), 7.34 (dd, 2H, *^3^J* = 8.6 Hz, *^3^J* = 7.6 Hz, H(Np)), 7.42 (dd, 2H, *^3^J* = 8.5 Hz, *^3^J* = 7.6 Hz, H(Np)), 7.77 (d, 2H, *^3^J* = 9.0 Hz, H(Np)), 7.84 (d, 2H, *^3^J* = 8.0 Hz, H(Np)), 8.10 (dd, 2H, *^3^J* = 7.3 Hz, *^4^J* = 1.0 Hz, H2? H2′(Dans)), 8.15 (d, 2H, *^3^J* = 8.7 Hz, H8, H8′(Dans)), 8.55 (d, 2H, *^3^J* = 8.4 Hz, H4, H4′(Dans)). ^13^C-NMR (100.6 MHz, CDCl_3_): δ 45.4 (4CH_3_N), 49.9 (2CH_2_N), 58.5 (2CH_3_O), 69.9 (2CH_2_O), 115.2 (2CH(Ar)), 116.5 (2C(Ar)), 117.4 (2CH(Ar)), 119.0 (2CH(Ar)), 119.7 (2CH(Ar)), 120.1 (2C(Ar)), 122.7 (2CH(Ar)), 123.1 (2CH(Ar)), 123.7 (2CH(Ar)), 124.3 (2CH(Ar)), 127.0 (2CH(Ar)), 127.7 (2CH(Ar)), 128.2 (2CH(Ar)), 129.5 (6CH(Ar)), 130.2 (2C(Ar)), 130.3 (2CH(Ar)), 130.7 (2CH(Ar)), 133.6 (2C(Ar)), 134.3 (2C(Ar)), 139.4 (2NC(Ar)), 140.0 (2NC(Ar)), 143.0 (2NC(Ar)), four quaternary carbon atoms were not assigned. MALDI-TOF calcd for C_62_H_61_N_6_O_6_S_2_ [M + H]^+^ 1049.4094, found: 1049.4030.

**Compound 23**. A one-neck flask equipped with a magnetic stirrer was charged with Compound **10** (87 mg, 0.15 mmol), acetonitrile (1 mL), 4-bromo-7-methoxycoumarin **12** (82 mg, 0.3 mmol), and K_2_CO_3_ (103 mg, 0.75 mmol), and the reaction mixture was stirred for 8 h at 50 °C. The residue was filtered, washed with dichloromethane (3 × 5 mL), and the combined organic fractions were evaporated in vacuo. Target Compound **23** was obtained as a brownish crystalline Compound. Yield 141 mg (98%), m.p. 126–128 °C. ^1^H-NMR (400 MHz, CDCl_3_): δ 3.28 (s, 6H, CH_3_O), 3.48–3.52 (m, 4H, CH_2_N), 3.53–3.56 (m, 4H, CH_2_O), 3.83 (s, 6H, CH_3_O), 4.56 (br. s, 4H, CoumCH_2_N), 5.47 (br. s, 2H, NH), 6.09 (s, 2H, =CH), 6.11 (t, 2H, *^4^J* = 2.0 Hz, H2(Ph)), 6.20 (dd, 2H, *^3^J* = 8.2 Hz, *^4^J* = 2.0 Hz, H(Ph)), 6.34 (dd, 2H, *^3^J* = 7.9 Hz, *^4^J* = 2.0 Hz, H(Ph)), 6.79 (dd, 2H, *^3^J* = 8.8 Hz, *^4^J* = 2.6 Hz, H8, H8′(Coum)), 6.82 (d, 2H, *^4^J* = 2.6 Hz, H6, H6′(Coum)), 6.97 (t, 2H, *^3^J* = 8.1 Hz, H5, H5′(Ph)), 7.03 (d, 2H, *^3^J* = 8.4 Hz, H3, H3′(BiNp)), 7.15 (ddd, 2H, *^3^J* = 8.9 Hz, *^3^J* = 6.9 Hz, *^4^J* = 1.2 Hz, H6, H6′(BiNp)), 7.24 (ddd, 2H, *^3^J* = 8.9 Hz, *^3^J* = 6.9 Hz, *^4^J* = 1.2 Hz, H7, H7′(BiNp)), 7.40 (d, 2H, *^3^J* = 8.8 Hz, H5, H5′(Coum)), 7.57 (d, 2H, *^3^J* = 8.9 Hz, H8, H8′(BiNp)), 7.74 (d, 2H, *^3^J* = 8.4 Hz, H4, H4′(BiNp)), 7.76 (d, 2H, *^3^J* = 8.9 Hz, H5, H5′(BiNp)). ^13^C-NMR (100.6 MHz, CDCl_3_): δ 50.9 (2CH_2_N), 51.8 (2CH_3_O), 55.6 (2CH_3_O), 58.9 (2CoumCH_2_N), 70.7 (2CH_2_O), 101.0 (2CH=), 103.8 (2CH(Ar)), 106.2 (2CH(Ar)), 109.0 (2CH(Ar)), 109.3 (2CH(Ar)), 111.4 (C4a, C4a′(Coum)), 112.1 (2CH(Ar)), 116.8 (2C(BiNp)), 118.7 (2CH(Ar)), 123.2 (2CH(Ar)), 124.1 (2CH(Ar)), 124.4 (2CH(Ar)), 126.7 (2CH(Ar)), 128.0 (2CH(Ar)), 129.0 (2CH(Ar)), 129.3 (2C(BiNp)), 129.9 (2CH(Ar)), 133.8 (2C(Ar)), 140.4 (2C(Ar)), 143.9 (2NC(Ar)), 148.2 (2NC(Ar)), 151.7 (2NC(Ar)), 155.5 (2CO), 161.2 (2CO), 162.5 (2CO). MALDI-TOF calcd for C_60_H_55_N_4_O_8_ [M + H]^+^ 959.4020, found: 959.3957.

**Compound 24**. A one-neck flask equipped with a magnetic stirrer was charged with Compound **10** (87 mg, 0.15 mmol), acetonitrile (1 mL), 4-bromo-6, 7-dimethoxycoumarin **20** (91 mg, 0.3 mmol), and K_2_CO_3_ (103 mg, 0.75 mmol), and the reaction mixture was stirred for 8 h at 50 °C. The residue was filtered, washed with dichloromethane (3 × 5 mL), and the combined organic fractions were evaporated in vacuo. Target Compound **24** was obtained as a brownish crystalline Compound. Yield 150 mg (98%), m.p. 131–133 °C. ^1^H-NMR (400 MHz, CDCl_3_): δ 3.29 (s, 6H, CH_3_O), 3.48–3.52 (m, 4H, CH_2_N), 3.53–3.57 (m, 4H, CH_2_O), 3.80 (s, 6H, CH_3_O), 3.87 (s, 6H, CH_2_O), 4.59 (br. s, 4H, CoumCH_2_N), 5.59 (br. s, 2H, NH), 6.10 (s, 2H, =CH), 6.16 (t, 2H, *^4^J* = 2.1 Hz, H2, H2′(Ph)), 6.20 (dd, 2H, *^3^J* = 8.2 Hz, *^4^J* = 2.1 Hz, H(Ph)), 6.38 (dd, 2H, *^3^J* = 7.8 Hz, *^4^J* = 2.1 Hz, H(Ph)), 6.77 (s, 2H, H(Coum)), 6.82 (s, 2H, H(Coum)), 6.96 (t, 2H, *^3^J* = 8.1 Hz, H5, H5′(Ph)), 7.02 (d, 2H, *^3^J* = 8.4 Hz, H3, H3′(BiNp)), 7.12 (ddd, 2H, *^3^J* = 8.8 Hz, *^3^J* = 6.8 Hz, *^4^J* = 1.1 Hz, H6, H6′(BiNp)), 7.20 (ddd, 2H, *^3^J* = 9.0 Hz, *^3^J* = 6.8 Hz, *^4^J* = 1.0 Hz, H7, H7′(BiNp)), 7.60 (d, 2H, *^3^J* = 9.0 Hz, H8, H8′(BiNp)), 7.73 (d, 4H, *^3^J_obs_* = 8.8 Hz, H4, H4′, H5, H5′(BiNp)). ^13^C-NMR (100.6 MHz, CDCl_3_): δ 50.7 (2CH_2_N), 51.9 (2CH_3_O), 56.1 (2CH_3_O), 56.2 (2CH_3_O), 58.9 (2CoumCH_2_N), 70.7 (2CH_2_O), 100.0 (2CH=), 103.8 (2CH(Ar)), 103.9 (2CH(Ar)), 106.1 (2CH(Ar)), 108.7 (2CH(Ar)), 109.6 (2CH(Ar)), 110.2 (C4a, C4a′(Coum)), 116.7 (2C(BiNp)), 118.5 (2CH(Ar)), 123.1 (2CH(Ar)), 124.3 (2CH(Ar)), 126.6 (2CH(Ar)), 127.9 (2CH(Ar)), 128.9 (2CH(Ar)), 129.2 (2C(BiNp)), 129.8 (2CH(Ar)), 133.8 (2C(Ar)), 140.2 (2C(Ar)), 143.8 (2NC(Ar)), 145.9 (2NC(Ar)), 148.3 (2NC(Ar)), 149.5 (2CO), 151.4 (2CO), 152.5 (2CO), 161.3 (2CO). MALDI-TOF calcd for C_62_H_59_N_4_O_10_ [M + H]^+^ 1019.4231, found: 1019.4285.

**Compound 25**. A two-neck flask equipped with a magnetic stirrer and reflux condenser, flushed with dry argon, was charged with Compound **10** (91 mg, 0.16 mmol), Pd(dba)_2_ (15 mg, 16 mol%), DavePhos (11 mg, 18 mol%), and absolute dioxane (2 mL). After stirring for 2 min, 6-bromoquinoline **12** (129 mg, 0.62 mmol) and *t*BuONa (74 mg, 0.77 mmol) were added and the reaction mixture was refluxed for 15 h. After the reaction was over, the residue was filtered, washed with dichloromethane (3 × 5 mL), combined filtrates were evaporated in vacuo, and the residue was chromatographed on silica gel using a sequence of eluents: CH_2_Cl_2_, CH_2_Cl_2_–MeOH 200:1–100:1. Target Compound **25** was obtained with CH_2_Cl_2_–MeOH 200:1 eluent. Yield 91 mg (71%), yellow crystalline Compound, m.p. 106–108 °C. ^1^H-NMR (400 MHz, CDCl_3_): δ 3.30 (s, 6H, CH_3_), 3.56 (t, 4H, *^3^J* = 6.1 Hz, CH_2_N), 3.92 (t, 4H, *^3^J* = 6.1 Hz, CH_2_O), 5.60 (s, 2H, NH), 6.65 (dd, 2H, *^3^J* = 7.9 Hz, *^4^J* = 1.9 Hz, H(Ph)), 6.70. (dd, 2H, *^3^J* = 7.9 Hz, *^4^J* = 1.9 Hz, H(Ph)), 6.76 (t, 2H, *^4^J* = 1.9 Hz, H2, H2′(Ph)), 7.08 (d, 2H, *^3^J* = 8.1 Hz, H3, H3′(BiNp)), 7.10 (t, 2H, *^3^J* = 7.9 Hz, H5, H5′(Ph)), 7.16–7.20 (m, 2H, H6, H6′(BiNp)), 7.18 (d, 2H, *^4^J* = 2.6 Hz, H5, H5′(Quin)), 7.25–7.30 (m, 4H, H7, H7′(BiNp), H3, H3′(Quin)), 7.37 (dd, 2H, *^3^J* = 9.3 Hz, *^4^J* = 2.6 Hz, H7, H7′(Quin)), 7.70 (d, 2H, *^3^J* = 9.0 Hz, H8, H8′(BiNp)), 7.81 (d, 2H, *^3^J* = 8.1 Hz, H4, H4′(BiNp)), 7.85 (d, 2H, *^3^J* = 9.0 Hz, H5, H5′(BiNp)), 7.90 (d, 2H, *^3^J* = 9.3 Hz, H8, H8′(Quin)), 7.94 (d, 2H, *^3^J* = 8.3 Hz, H4, H4′(Quin)), 8.69 (dd, 2H, *^3^J* = 4.1 Hz, *^4^J* = 0.9 Hz, H2, H2′(Quin)). ^13^C-NMR (100.6 MHz, CDCl_3_): δ 51.8 (2CH_2_N), 59.0 (2CH_2_O), 69.4 (2CH_2_O), 112.3 (2CH(Ar)), 114.0 (2CH(Ar)), 114.2 (2CH(Ar)), 116.6 (2CH(Ar)), 116.8 (2C(Ar)), 118.1 (2CH(Ar)), 121.2 (2CH(Ar)), 123.6 (2CH(Ar)), 124.4 (2CH(Ar)), 124.8 (2CH(Ar)), 127.0 (2CH(Ar)), 128.1 (2CH(Ar)), 129.3 (2CH(Ar)), 129.5 (4C(Ar)), 129.6 (2CH(Ar)), 130.1 (2CH(Ar)), 133.8 (2C(Ar)), 134.7 (2CH(Ar)), 139.8 (2C(Ar)), 143.7 (2C(Ar)), 143.9 (2C(Ar)), 145.8 (2C(Ar)), 147.3 (2CH(Ar)), 148.1 (2C(Ar)). MALDI-TOF calcd for C_56_H_49_N_6_O_2_ [M + H]^+^ 837.3917, found: 837.3882.

**Compound 26**. A two-neck flask equipped with a magnetic stirrer and reflux condenser, flushed with dry argon, was charged with Compound **10** (78 mg, 0.13 mmol), Pd(dba)_2_ (12 mg, 16 mol%), DavePhos (9 mg, 18 mol%), and absolute dioxane (2 mL). After stirring for 2 min, 6-bromoquinoline **21** (112 mg, 0.53 mmol) and *t*BuONa (64 mg, 0.67 mmol) were added and the reaction mixture was refluxed for 15 h. After the reaction was over, the residue was filtered, washed with dichloromethane (3 × 5 mL), combined filtrates were evaporated in vacuo, and the residue was chromatographed on silica gel using a sequence of eluents: CH_2_Cl_2_, CH_2_Cl_2_–MeOH 500:1–100:1. Target Compound **26** was obtained with CH_2_Cl_2_–MeOH 250:1 eluent. Yield 44 mg (39%), yellow viscous oil. ^1^H-NMR (400 MHz, CDCl_3_): δ 3.28 (s, 6H, CH_3_), 3.54 (t, 4H, *^3^J* = 5.7 Hz, CH_2_O), 3.90 (t, 4H, *^3^J* = 5.7 Hz, CH_2_N), 5.56 (s, 2H, NH), 6.64 (d, 2H, *^3^J* = 8.7 Hz, H(Ph)), 6.66–6.70 (m, 4H, H(Ph)), 7.08 (d, 2H, *^3^J* = 8.3 Hz, H3, H3′(BiNp)), 7.09 (t, 2H, *^3^J* = 8.4 Hz, H5, H5′(Ph)), 7.17–7.21 (m, 2H, H6, H6′(BiNp)), 7.27–7.31 (m, 2H, H7, H7′(BiNp)), 7.47–7.56 (m, 4H, H6, H6′, H7, H7′ (Quin)), 7.63–7.67 (m, 4H, H4, H4′, H5, H5′(Quin)), 7.70 (d, 2H, *^3^J* = 9.0 Hz, H8, H8′(BiNp)), 7.82 (d, 2H, *^3^J* = 8.3 Hz, H4, H4′(BiNp)), 7.88 (d, 2H, *^3^J* = 8.8 Hz, H5, H5′(BiNp)), 8.10 (br. d, 2H, *^3^J_obs_* = 7.6 Hz, H8, H8,(Quin)), 8.64 (br. d, 2H, *^4^J* = 2.6 Hz, H2, H2′(Quin)). ^13^C-NMR (100.6 MHz, CDCl_3_): δ 51.9 (2CH_2_N), 59.1 (2CH_3_O), 69.5 (2CH_2_O), 113.4 (2CH(Ph)), 114.5 (2CH(Ph)), 116.0 (2CH(Ph)), 117.2 (2C(Ar)), 118.3 (2CH(Ar)), 122.0 (2CH(Ar)), 123.7 (2CH(Ar)), 124.5 (2CH(Ar)), 126.6 (2CH(Ar)), 127.1 (2CH(Ar)), 127.4 (2CH(Ar)), 127.5 (2CH(Ar)), 128.2 (2CH(Ar)), 129.0 (2CH(Ar)), 129.5 (2CH(Ar)), 129.6 (2CH(Ar)), 130.5 (2CH(Ar)), 133.8 (2CH(Ar)), 139.7 (4C(Ar)), 141.6 (4C(Ar)), 144.3 (2CH(Ar)), 147.2 (4C(Ar)). MALDI-TOF calcd for C_56_H_49_N_6_O_2_ [M + H]^+^ 837.3917, found: 837.3855.

Titrations of the selected Compounds with amino alcohols using the fluorescence spectra were conducted as follows: The spectrofluorometric cuvette (l = 1 cm) was charged with 3 mL of the 10^−5^ M solution of the corresponding ligand **7**, **14**–**16**, **22**–**26**, and an individual enantiomer of the certain amino alcohol (leucinol, *tert*-leucinol, valinol, 2-amino-1-propanol, phenylglycinol, 2-amino-1-butanol, 2-amino-1,2-diphenylethanol) (C = 0.2 M) was added stepwise (100, 200, 500, 1000 equiv.) into the cuvette. Emission spectra were recorded with each addition. The excitation wavelengths: 310 nm (**7, 16, 25**), 340 nm (**14**, **22**), 320 nm (**15**, **23**), 330 nm (**24**), and 350 nm (**26**), and they corresponded to the absorption maxima of the ligands under investigation.

## 3. Results and Discussion

### 3.1. Synthesis of (S)-BINAM-Containing Detectors

**Scheme 1:**
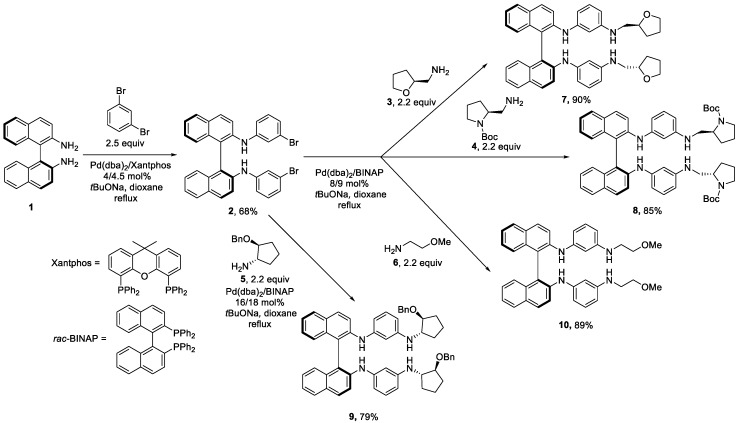
Pd-catalyzed modification of BINAM with *N*- and *O*-containing substituents.

The synthesis of the crucial intermediate, *N,N′*-di(3-bromophenyl)substituted (*S*)-BINAM, was accomplished according to a procedure developed by us earlier [41] by reacting free diamine **1** with a slight excess (2.2 equiv.) of 1,3-dibromobenzene in the presence of Pd(dba)_2_/Xantphos (4/4.5 mol%) catalyst (Scheme 1). 

Compound **2** was isolated in a 68% yield and introduced in further Pd(0)-catalyzed amination reactions. The first of them was carried out with (*S*)-tetrahydrofurfuryl amine (**3**) and resulted in a 90% yield of the target product of diamination **7**. This Compound as well as many others were obtained in the individual state by the column chromatography on silica gel. For this reaction and for subsequent syntheses, the Pd(dba)_2_/*rac*-BINAP catalytic system was used, which was shown previously to be the most versatile and convenient. The reactions with two other chiral amines **4** and **5** also produced corresponding diaminated products **8** and **9** in high yields; in the latter case, the use of 16 mol% catalyst was inevitable due to steric hindrances in the amine **5**. Compounds **7**–**9** differ by the nature of the donor atoms in the substituents, their reciprocal positions, and steric hindrances. They possess, besides the *C*2 chiral BINAM moiety, two (Compounds **7** and **8**) or four (derivative **9**) additional chiral centers, which may enhance their ability to distinguish between two enantiomers of the analyte. Lastly, simple 2-methoxyethylamino substituents were introduced in the diphenyl-BINAM framework in view of employing the only *C*2 chirality of the diaminobinaphthalene fragment in the perspective detector (synthesis of Compound **10**).

**Scheme 2:**
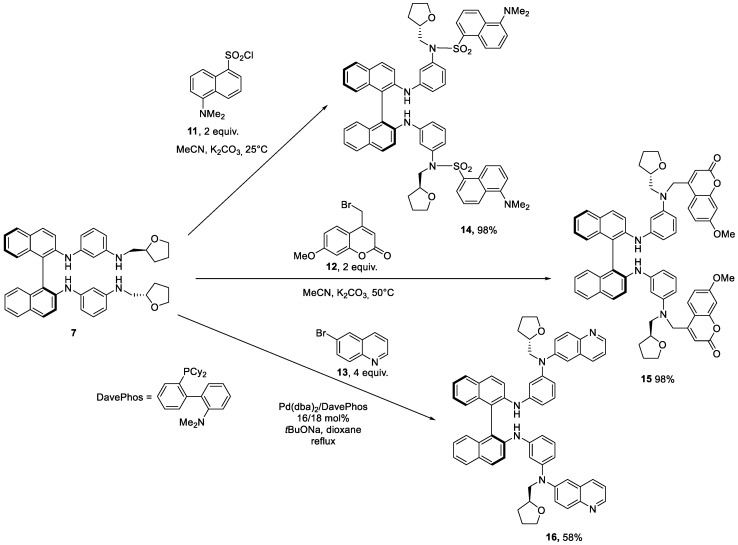
Modification of the Compound **7** with fluorophore groups.

At the next step, the Compound **7** possessing two tetrahydrofurfurylamino substituents was subjected to modifications with several fluorophore groups (Scheme 2). Such modifications resulted in the change of the emission maxima, shifting them from 460 nm (parent Compound **7**) to 505 (Compound possessing 6-aminoquinolinyl moieties) or 520 nm (Compound with dansylamide moieties). Additionally, the number of coordination sites increased after the modifications as all fluorophores employed possess additional *N* or *O* donor atoms. The reaction with dansyl chloride **11** allowed the didansylated product **14** in almost a quantitative yield, and this process was carried out at room temperature. The introduction of two 7-methoxycoumarin substituents was accomplished at a slightly elevated temperature to ensure full conversion of the starting Compounds, and the target derivative **15** was also isolated in a 98% yield by a simple work-up of the reaction mixture. The reaction with 6-bromoquinoline (**13**) proceeded under the catalytic conditions, where the application of 16% mol catalyst and 4 equiv. of the bromide was important to facilitate the heteroarylation of two secondary amino groups. More of the electron-donating DavePhos ligand was used instead of BINAP in this coupling. The isolation of the target Compound **16** by column chromatography afforded a 58% yield of the diquinolinyl derivative. 

**Scheme 3:**
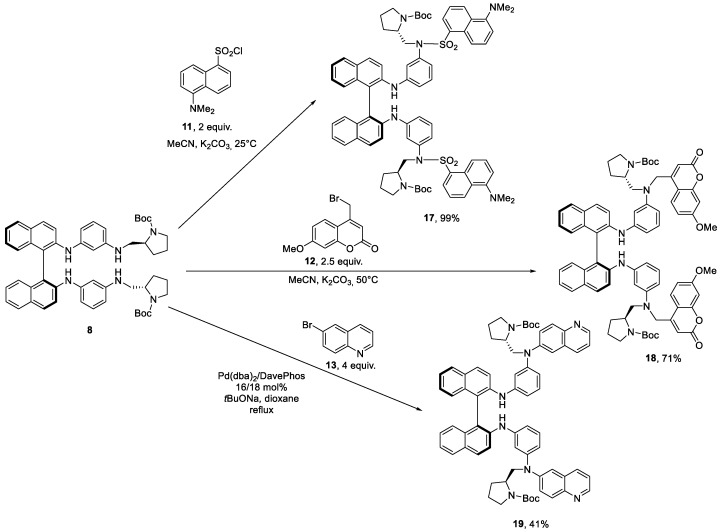
Modification of the Compound **8** with fluorophore groups.

The BINAM derivative **8** with two *N*-Boc-substituted pyrrolidine moieties was reacted with dansyl chloride **11** and 4-bromomethyl)-7-methoxycoumarin **12** under similar conditions, giving the corresponding derivatives **17** and **18** in 99% and 71% yields, respectively (Scheme 3). It was also modified with the quinolin-6-yl fluorophore group. For this purpose, the reaction with 6-bromoquinoline (**13**) (4 equiv.) was accomplished under catalytic conditions to produce the desired product **19** in a 41% yield. For all derivatives **17**–**19**, the NMR spectra are quite complicated due to the presence of two *N*-Boc groups, resulting in four rotamers. The number of the signals substantially increases together with the line broadening of many of them. The influence of the *N*-Boc group is different for various parts of the molecule, more seriously altering the aliphatic part. 

The decoration of the BINAM derivative **9** containing two benzyloxy-substituted cyclopentylamino groups with additional fluorophore groups was impossible due to steric hindrances at the secondary amino groups. Neither the reaction with dansyl chloride, even at an elevated temperature, nor the attempts to introduce the 7-methoxycoumarin fluorophore substituent were successful. Thus, it should be employed in the fluorescence studies as it is. 

**Scheme 4:**
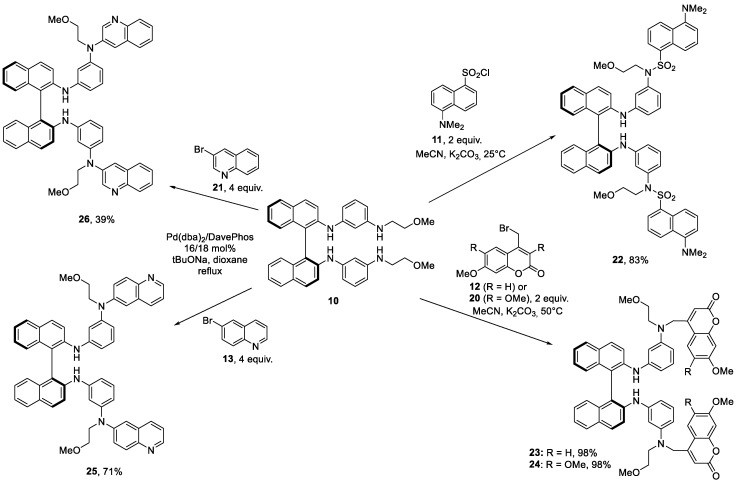
Modification of the Compound **10** with fluorophore groups.

The BINAM derivative **10** was introduced in the reactions with five Compounds to ensure the variety of the fluorophore substituents (Scheme 4). While the didansyl derivative **22** was obtained in an 83% yield, both 7-methoxy- and 6, 7-dimethoxycoumarin derivatives **23** and **24** were synthesized in almost quantitative yields. The catalytic reaction with 6-bromoquinoline (**13**) was more successful (71% yield of **25**) than that with the isomeric 3-bromoquinoline (**21**) (39% yield of **26**) because in the latter case, two successive chromatographic purifications were needed to separate the excess of the starting bromide. The necessity of obtaining such isomers in which quinoline groups are attached in a different manner to the BINAM-based framework is due not only to a difference in the spectral properties on 6- and 3-aminoquinolines but also stems from the different positions of the nitrogen atoms in these two derivatives, which may result in the different binding modes of these molecules.

### 3.2. Spectroscopic Investigations

The recognition of the optically active molecules by fluorescent detectors is based on the fact that the coordination of the chiral detector with an enantiomer of the chiral analyte leads to a formation of the chiral complex, and the coordination with the opposite enantiomer of the analyte results in another complex. These complexes, being diastereomers, may possess different fluorescence. For the qualitative analysis, the preferable case is the change of the fluorescence in the presence of one enantiomer and the absence of any change or the change with the opposite sign of the fluorescence in the presence of the opposite enantiomer. More interesting, however, and rarer is the shift of the emission maximum upon the addition of the enantiomer. In the majority of cases, the molecular complexes are formed via hydrogen bonds, and π interactions are also possible. 

**Figure 1:**
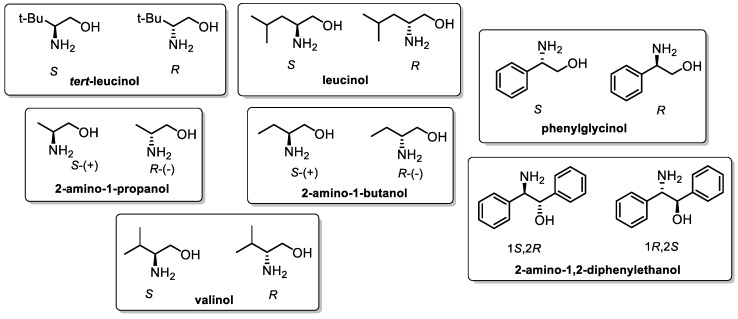
Amino alcohols employed in this work.

The investigation of the ability of synthesized Compounds to act as fluorescent enantioselective detectors was carried out using a panel of seven amino alcohols differing by the nature of the substituents (Figure 1). In principle, the most demanding analytes to be detected by means of the fluorescence spectra are amino acids and some α-hydroxy acids. For the sake of the simplicity of the experimental procedures, the corresponding amino alcohols or diols can be used instead of the acids as the latter do not dissociate, are well-soluble in the majority of solvents, and do not need the control of the media pH. Each amino alcohol was tested as an individual enantiomer; 100, 200, 500, and 1000 equiv. of each enantiomer were added successively to a solution of selected BINAM derivatives and the fluorescence spectra were recorded. Such a great excess of amino alcohols was necessary due to the obviously low binding constants and the need to observe a reliable spectroscopic response of the system. All experiments were conducted in MeCN at the ligand concentrations of ca. 10^−5^ M and 0.2 M concentrations of amino alcohols. Selected data are discussed, which show the influence of the ligands’ structures on their sensing properties and only significant data are presented, where the recognition of the enantiomers was possible. Plausible coordination patterns for amino alcohols with our ligands are shown in Supplementary Materials Figure S1.

**Figure 2:**
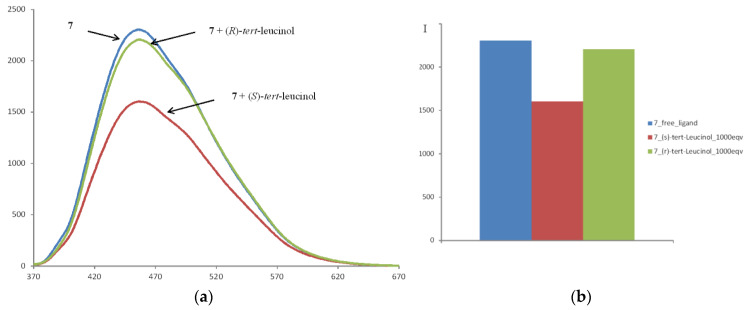
Fluorescence spectra of Compound **7** in the presence of (*R*)- and (*S*)-enantiomers of *tert*-leucinol (1000 equiv.); (**a**) general view; (**b**) schematic representation of the changes in emission.

**Figure 3:**
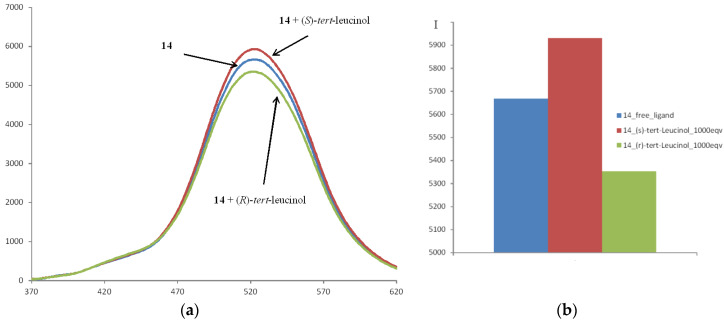
Fluorescence spectra of Compound **14** in the presence of (*R*)- and (*S*)-enantiomers of *tert*-leucinol (1000 equiv.); (**a**) general view; (**b**) schematic representation of the changes in emission.

The first studied Compound was **7** possessing two tetrahydrofurfurylamino moieties. The BINAM fragment is characterized by the absorption maximum at 310 nm and emits at 460 nm. The addition of (*S*)-*tert*-leucinol resulted in emission quenching while the presence of the (*R*)-isomer almost did not change the fluorescence spectrum (Figure 2). The addition of other amino alcohol either did not change the spectra at all, and both enantiomers led to similar and quite small changes in the emission. The modification of this ligand with two dansyl groups (Compound **14**) resulted in the changes of the absorption and emission maxima (340 and 525 nm, respectively); however, it made the ligand’s fluorescence less sensitive to the presence of the amino alcohols. Only in the case of the same *tert*-leucinol can one note small changes with the opposite sign in the emission spectrum in the presence of two enantiomers (Figure 3). Such weak changes disfavor its sensing abilities.

**Figure 4:**
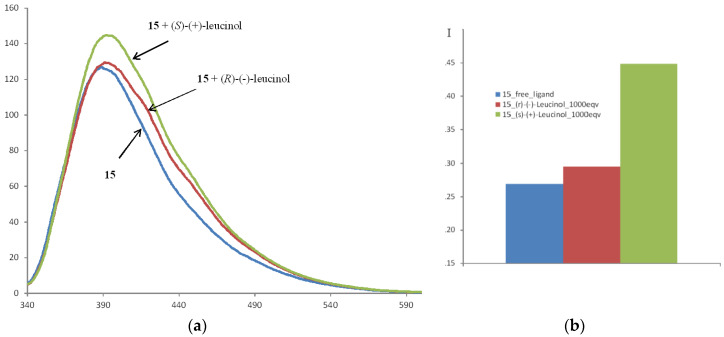
Fluorescence spectra of Compound **15** in the presence of (*R*)- and (*S*)-enantiomers of leucinol (1000 equiv.); (**a**) general view; (**b**) schematic representation of the changes in emission.

**Figure 5:**
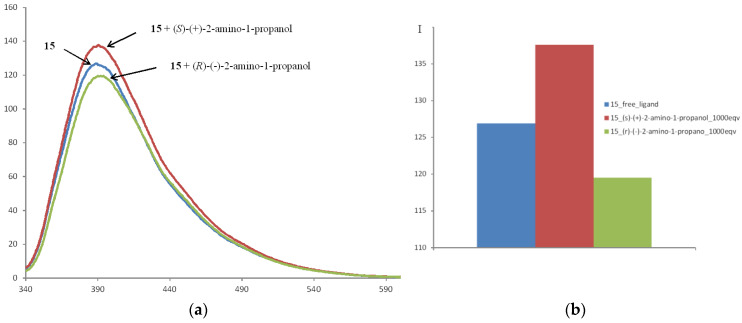
Fluorescence spectra of Compound **15** in the presence of (*R*)- and (*S*)-enantiomers of 2-amino-1-propanol (1000 equiv.); (**a**) general view; (**b**) schematic representation of the changes in emission.

Changing the dansyl fluorophore groups for 7-methoxycoumarin substituents (Compound **15**) resulted in the absorption at 320 nm and the emission maximum shifted to the shortwave region at 390 nm (Figure 4). The addition of (*S*)-leucinol slightly increased the fluorescence intensity while (*R*)-isomer did not change it at all. Like its dansylated analog **14**, 7-methoxycoumarin derivative **15** in other cases showed weak responses to the addition of other amino alcohols, e.g., the two enantiomers of 2-amino-1-propanol only slightly changed its emission intensity (Figure 5).

**Figure 6:**
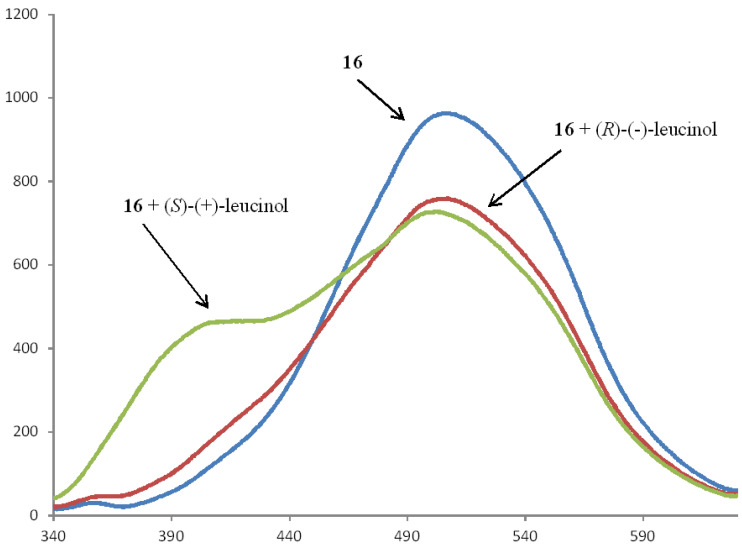
Fluorescence spectra of Compound **16** in the presence of (*R*)- and (*S*)-enantiomers of leucinol (1000 equiv.).

**Figure 7:**
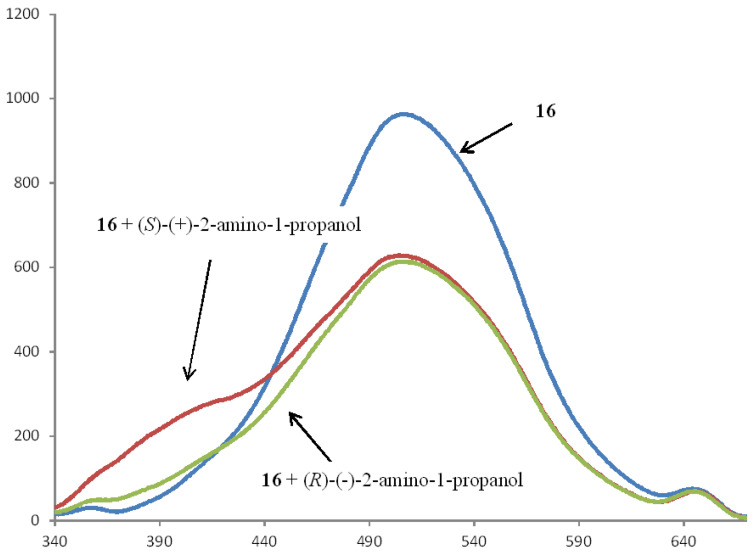
Fluorescence spectra of Compound **16** in the presence of (*R*)- and (*S*)-enantiomers of 2-amino-1-propanol (1000 equiv.).

The ligand **16** possesses two 6-aminoquinoline moieties, with the emission maximum at 505 nm. The addition of (*S*)- and (*R*)-leucinol led to emission quenching, but in the case of the (*S*)-isomer, the second maximum of lower intensity emerged at 415 nm (Figure 6); thus, it can be used for recognition between these two enantiomers. A similar spectral behavior of Compound **16** was noted when adding (*S*)-2-amino-1-propanol (Figure 7); however, in this case, the effect was not so pronounced as only the shoulder at 400–420 nm was noted. The nature of these additional emission maxima in the spectra is vague. (*S*)-enantiomers of *tert*-leucinol and 2-amino-1-propanol did not cause this effect with the other ligands tested, and one may assume that this effect could arise from a specific interaction of two fluorophore moieties present in the Compound 16, i.e., BINAM and quinoline fragments. The coordination with these aminoalcohols could promote the rapprochement of the two fluorophores to produce a new emission band. However, this phenomenon needs to be studied specially. The second plausible explanation is the coordination of the amino alcohol with the nitrogen atom of the quinoline moiety causing the hypsochromic shift. Another coordination mode that does not involve the fluorophore group simply leads to fluorescence quenching. (*R*)-isomer participates only in the second type of coordination due to the different stabilities of the diastereomers of the molecular complexes.

**Figure 8:**
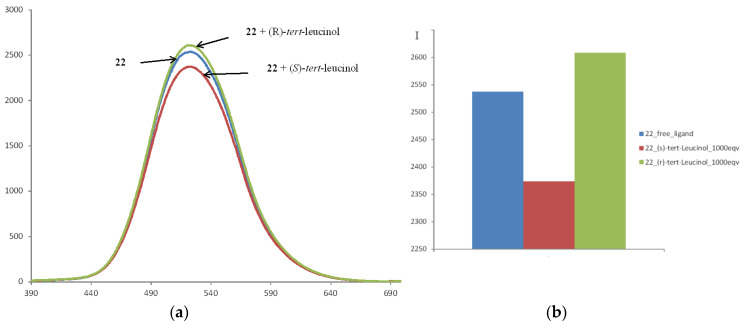
Fluorescence spectra of Compound **22** in the presence of (*R*)- and (*S*)-enantiomers of *tert*-leucinol (500 equiv.); (**a**) general view; (**b**) schematic representation of the changes in emission.

**Figure 9:**
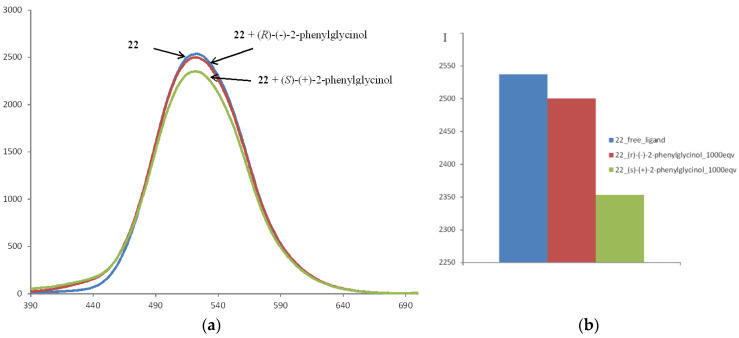
Fluorescence spectra of Compound **22** in the presence of (*R*)- and (*S*)-enantiomers of 2-phenylglycinol (1000 equiv.); (**a**) general view; (**b**) schematic representation of the changes in emission.

Next, we studied the BINAM derivatives bearing two 2-methoxyethylamino substituents. The Compound **22** possessing two additional dansyl fluorophore groups was not informative enough as upon the addition of the amino alcohols, the changes in its emission spectrum were tiny. It could hardly recognize the different enantiomers of *tert*-leucinol (Figure 8) and 2-phenylglycinol (Figure 9): In each case, (*S*)-isomer slightly quenched the emission while (*R*)-isomer did not change it at all. One may assume that two dansyl groups are spacious enough and shield the chiral centers of the ligand, hindering the formation of the molecular complexes of **14** and **22** with amino alcohols. A similar behavior of the dansylated derivatives of chiral cryptands was observed by us earlier [44].

**Figure 10:**
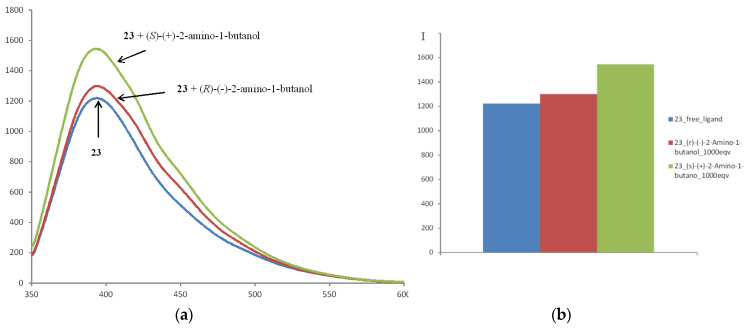
Fluorescence spectra of Compound **23** in the presence of (*R*)- and (*S*)-enantiomers of 2-amino-1-butanol (1000 equiv.); (**a**) general view; (**b**) schematic representation of the changes in emission.

**Figure 11:**
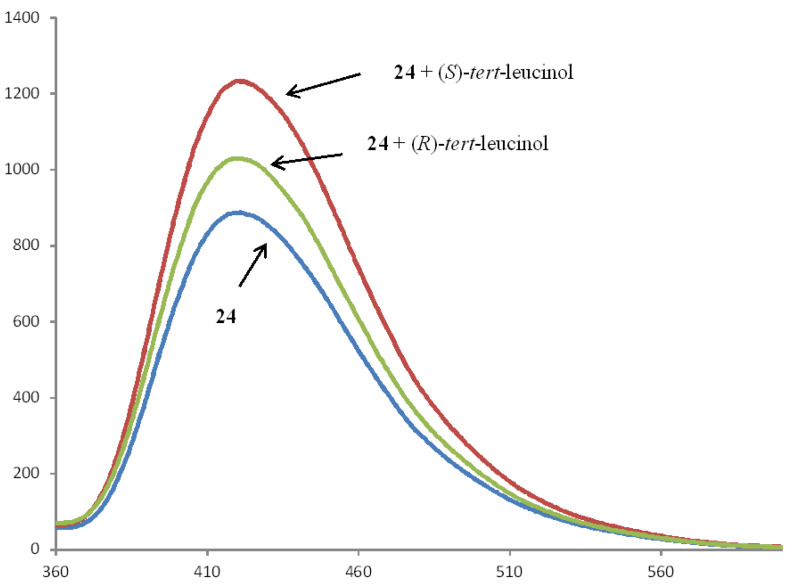
Fluorescence spectra of Compound **24** in the presence of (*R*)- and (*S*)-enantiomers of *tert*-leucinol (1000 equiv.).

**Figure 12:**
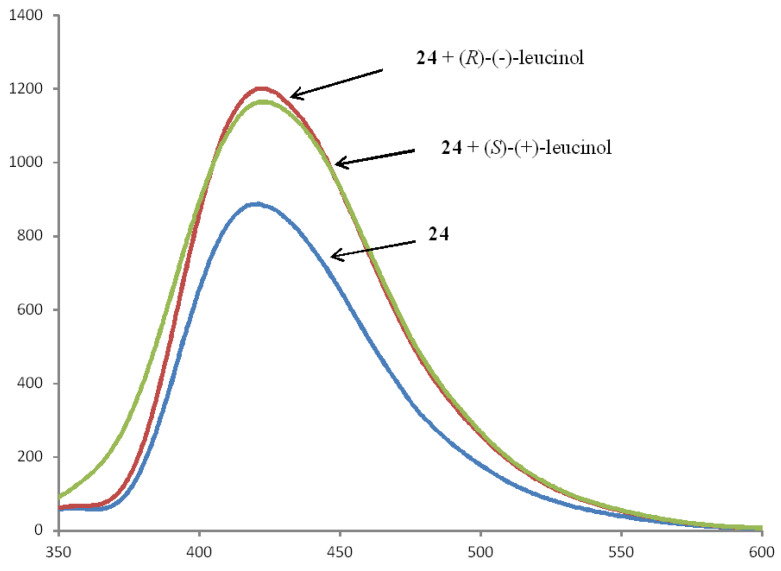
Fluorescence spectra of Compound **24** in the presence of (*R*)- and (*S*)-enantiomers of leucinol (1000 equiv.).

The introduction of 7-methoxy- and 6, 7-dimethoxycoumarin fluorophore groups in the parent molecule of **10**, resulting in the ligands **23** and **24**, changed the spectral response of these Compounds to the presence of amino alcohols. In all cases, a certain enhancement of the fluorescence upon the addition of the analytes was observed, though only in one case, in a pair **23**/2-amino-1-butanol, was the difference in this increase substantial for the opposite enantiomers (Figure 10). The addition of (*S*)-isomer resulted in much more pronounced emission enhancement than that of (*R*)-isomer. In the case of **24**, the difference for the opposite enantiomers of any amino alcohol was less significant (e.g., with *tert*-leucinol, Figure 11). With some other analytes, there was no difference in the spectroscopic response at all, e.g., in the case of leucinol enantiomers (Figure 12). Though the alteration of the emission by opposite enantiomers differing in the intensity but with the same sign is not suitable for qualitative observations, it can be employed for quantification, i.e., control of the optical purity of the analyte, provided the changes are substantial and well distinguishable.

**Figure 13:**
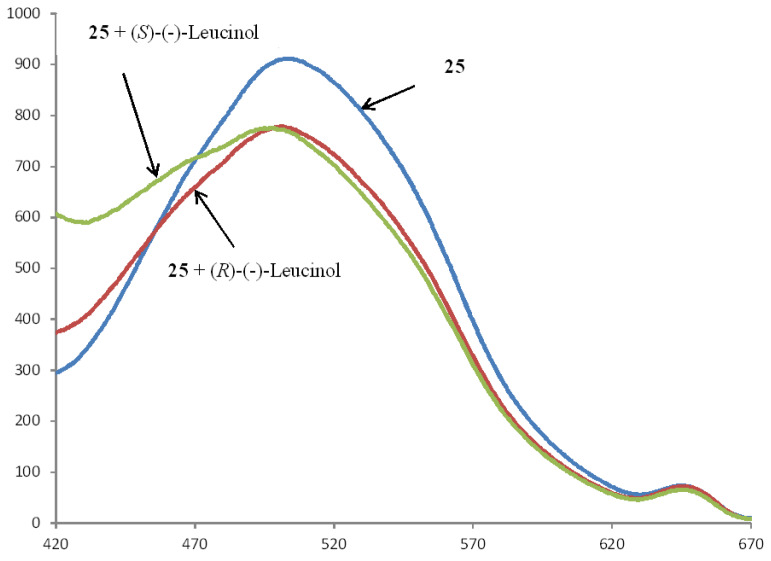
Fluorescence spectra of Compound **25** in the presence of (*R*)- and (*S*)-enantiomers of leucinol (1000 equiv.).

Compound **25** possesses two 6-aminoquinoline fluorophore groups and the emission maximum at 505 nm (Figure 13). The addition of both (*S*)-and (*R*)-leucinol led to an equal emission quenching at this wavelength. 

**Figure 14:**
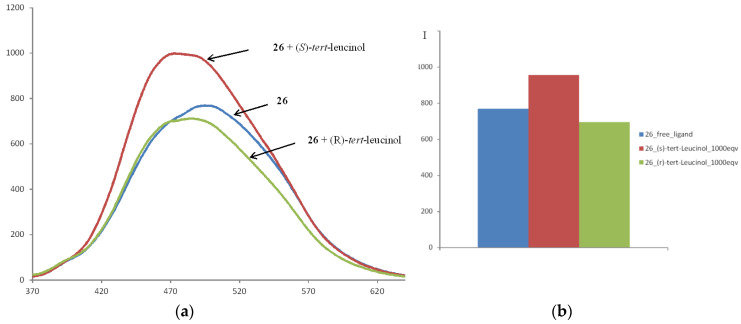
Fluorescence spectra of Compound **26** in the presence of (*R*)- and (*S*)-enantiomers of *tert*-leucinol (1000 equiv.); (**a**) general view; (**b**) schematic representation of the changes in emission.

**Figure 15:**
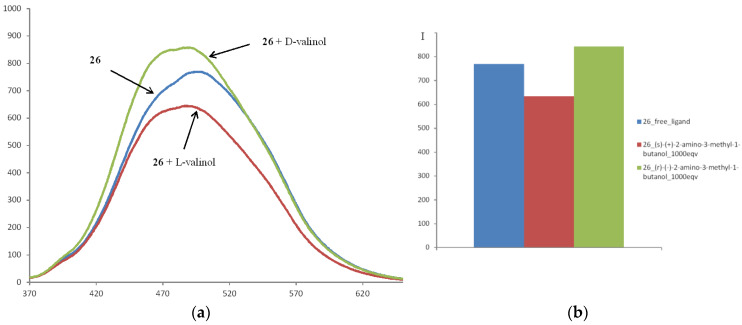
Fluorescence spectra of Compound **26** in the presence of L- and D-valinol (1000 equiv.); (**a**) general view; (**b**) schematic representation of the changes in emission.

**Figure 16:**
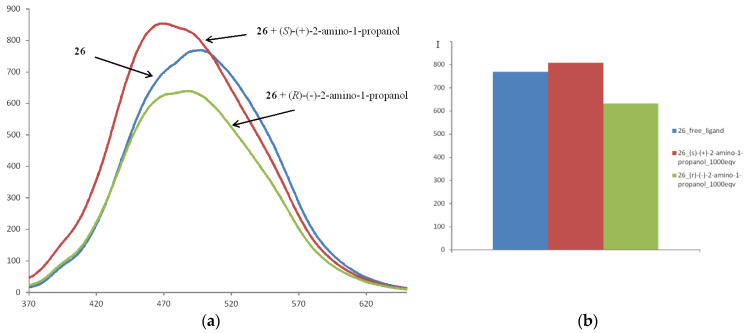
Fluorescence spectra of Compound **26** in the presence of (*R*)- and (*S*)-enantiomers of 2-amino-1-propanol (1000 equiv.); (**a**) general view; (**b**) schematic representation of the changes in emission.

The introduction of the isomeric 3-aminoquinoline fluorophore in the ligand **26** was efficient enough. The Compound is able to distinguish between the enantiomers of *tert*-leucinol (Figure 14), valinol (Figure 15), and 2-amino-1-propanol (Figure 16). The ligand itself possesses the emission maximum at 500 nm, and the addition of (*S*)-*tert*-leucinol resulted in the increase in the intensity of the fluorescence with a simultaneous hypsochromic shift of the maximum by *ca* 20 nm. In the presence of (*R*)-isomer, a similar shift was also observed together with a tiny quenching of the emission.

With D-valinol, the emission enhancement was achieved while with L-valinol promoted the emission quenching. At last, (*S*)- and (*R*)-enantiomers of 2-amino-1-propanol changed the intensity of fluorescence with opposite signs, and with this amino alcohol, the hypsochromic shift was the most prominent (up to 30 nm). In all experiments, except for the titrations with the Compound **26**, we observed changes in the intensities of fluorescence. Such behavior is usual in the case when the photoinduced electron transfer (PET) mechanism of fluorescence is applicable [46]. Only in the case of **26** were the hypsochromic shifts of the emission maximum noted upon the addition of the amino alcohols, which may imply that in these cases, the photoinduced charge transfer (PCT) mechanism might be considered. This mechanism is well documented for such fluorophore groups like dansyl, coumarin, and quinoline which possess electron-donating and electron-withdrawing sites to achieve the charge transfer in the fluorophore moiety. This mechanism demands that such an interaction with the analyte takes place, which alters this intramolecular charge transfer. In the case of **26**, the quinoline nitrogen atom is situated closer to the methoxy group in the substituent and may participate in the binding of the amino alcohol through the H-bond (Supplementary Materials Figure S2). 

We chose *tert*-leucinol to follow the response of all our detectors after the addition of its enantiomers. We showed above that the Compounds **7**, **14**, **22**, and **26** are able to recognize its enantiomers while the Compound **24** can be used only in quantitative assignments because both enantiomers led to emission enhancement (Figure 11). In the case of the ligand **16**, both enantiomers quenched fluorescence, but in a different way (Figure S4), as the coumarin-containing Compound **15** did not change its fluorescence at all in the presence of *tert*-leucinol (Figure S3). While ligand **23** responded to both enantiomers by equal intensification of the emission (Figure S5), the fluorescence of the Compound **25** was equally quenched by both enantiomers (Figure S6). 

The response of the most interesting detector **26** upon the addition of all amino alcohols can also be mentioned. It cannot distinguish between enantiomers of leucinol (Figure S7), 2-phenylglycinol (Figure S8), or 2-amino-1,2-diphenylethanol (Figure S10) as both enantiomers of these analytes gave rise to more or less emission enhancement with simultaneous hypsochromic shifts of the maximum by 10–30 nm. The recognition of 2-amino-1-butanol (Figure S9) is dubious as the quenching with its (*S*)-isomer is very tiny. Thus, we can conclude that the best detector is able to recognize enantiomers of no more than three amino alcohols. It means that even small changes in the nature of the substituents in these analytes (say, a change of methyl for ethyl) seriously affect the possibility of forming a molecular complex with the detector to promote necessary changes in the fluorescence.

## 4. Conclusions

As a result of the research, a new family of (*S*)-BINAM-based potential enantioselective fluorescent detectors were synthesized. Pd(0)-catalyzed amination was successfully employed for the introduction of chiral *N*- and *N,O*-containing substituents and additional fluorophore groups. This methodology allowed the synthesis of Compounds combining the *C*2 chirality of the central BINAM structural unit and an additional two or four stereocenters. The introduction of such fluorophore groups like dansyl and aminoquinoline led to emission at larger wavelengths compared to BINAM. In addition, the number of donor *N* and *O* atoms able to participate in binding analytes was increased by such modifications. Nine selected Compounds were tested for their ability to act as enantioselective fluorescent detectors of amino alcohols. Compounds **7**, **16**, **25**, and especially **26** were found to recognize the enantiomers of some analytes by specific changes in their emission spectra, and Compounds **15** and **23** also demonstrated a different behavior in the presence of some amino alcohols, but the changes in the spectra of fluorescence were tiny enough. Compound **26** possessing two 3-aminoquinolines is the most versatile as it was shown to recognize enantiomers of *tert*-leucinol, valinol, and 2-amino-1-propanol. Among the amino alcohols tested, *tert*-leucinol was most often the analyte that could be detected by the tested Compounds. It may be due to the presence of a bulky *tert*-butyl group in its structure, as it is well known that stereodiscrimination is favored by bulky substituents in interacting species. Enantiomers of less bulky leucinol could be recognized in fewer cases. 

## Supplementary Materials

The following are available online at https://www.mdpi.com/1424-8220/20/11/3234/s1, Figure S1: Plausible coordination patterns for amino alcohols with the BINAM-based ligands, Figure S2: Plausible coordination of amino alcohols with the ligand **26**, Figure S3: Fluorescence spectra of Compound **15** in the presence of (*R*)- and (*S*)-enantiomers of *tert*-leucinol (1000 equiv.), Figure S4: Fluorescence spectra of Compound **16** in the presence of (*R*)- and (*S*)-enantiomers of *tert*-leucinol (1000 equiv.), Figure S5: Fluorescence spectra of Compound **23** in the presence of (*R*)- and (*S*)-enantiomers of *tert*-leucinol (1000 equiv.), Figure S6: Fluorescence spectra of Compound **25** in the presence of (*R*)- and (*S*)-enantiomers of *tert*-leucinol (1000 equiv.), Figure S7: Fluorescence spectra of Compound **26** in the presence of (*R*)- and (*S*)-enantiomers of leucinol (1000 equiv.), Figure S8: Fluorescence spectra of Compound **26** in the presence of (*R*)- and (*S*)-enantiomers of 2-phenylglycinol (1000 equiv.), Figure S9: Fluorescence spectra of Compound **26** in the presence of (*R*)- and (*S*)-enantiomers of 2-amino-1-butanol (1000 equiv.), Figure S10: Fluorescence spectra of Compound **26** in the presence of the enantiomers of 2-amino-1,2-diphenylethanol (1000 equiv.).
