# Supplementary material for: Synthesis and Evaluation of the (S)-BINAM Derivatives as Fluorescent Enantioselective Detectors
Source: Sensors (Basel). 2020 Jun 6;20(11):3234. doi: 10.3390/s20113234 (PMC7308982; doi:10.3390/s20113234)
Supplement: Supplementary file 1 [file sensors-20-03234-s001.pdf]

Synthesis and Evaluation of the (*S*)-BINAM Derivatives as Fluorescent Enantioselective Detectors

Alexander V. Shaferov, Anna S. Malysheva, Alexei D. Averin, Olga A. Maloshitskaya and Irina P. Beletskaya

Supporting Information

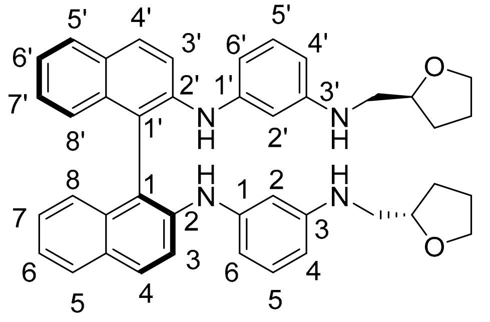

7

N1,N**$^{1'}$-((*S*)-[1,1'-binaphthalene]-2,2'-diyl)bis(*N*^{3}-(((S)-tetrahydrofuran-2yl)methyl)benzene-1,3-diamine) (7).**

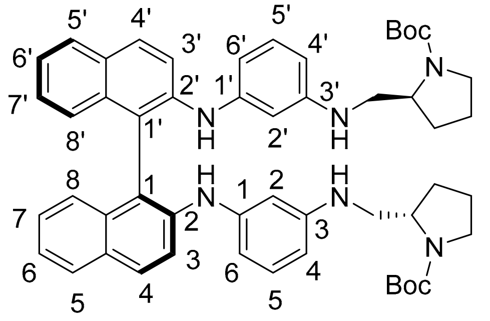

8

Di-*tert*-butyl 2,2'-((((((*S*)-[1,1'-binaphthalene]-2,2'-diyl)bis(azanediyl))bis(3,1-phenylene)bis.

(azanediyl))bis(methylene))(2*S*,2'*S*)-bis(pyrrolidine-1-carboxylate) (8).

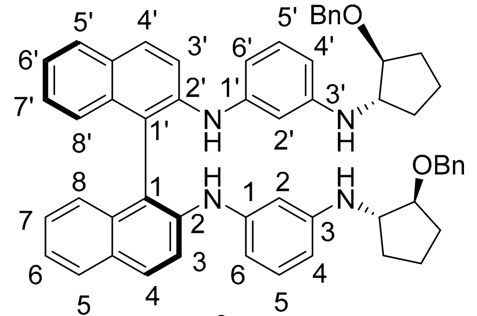

9

***N*^{1}*,N*$^{1'}$-((*S*)-[1,1'-binaphthalene]-2,2'-diyl)bis(*N*^{3}-((1*S*,2*S*)-2-(benzyloxy)cyclopentyl)benzene-1,3-diamine) (9).**

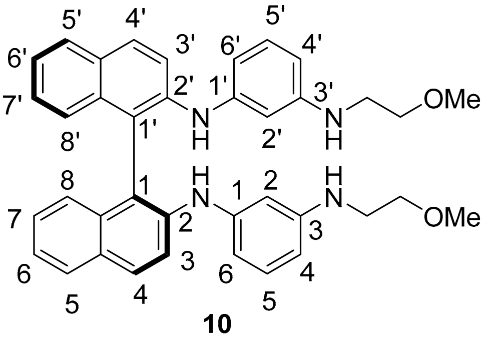

10

(*S*)-N^1,$N^{1'}$-([1,1'-binaphthalene]-2,2'-diyl)bis(N^3-(2-methoxyethyl)benzene-1,3-diamine) (10).

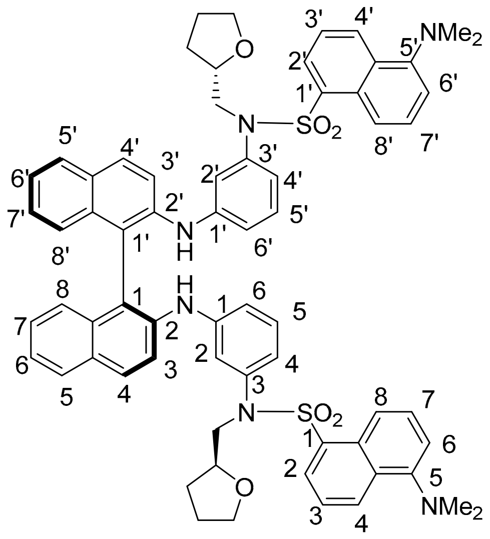

14

5-(Dimethylamino)-*N*-(3-(((*S*)-2'-((3-((5-(dimethylamino)-*N*-(((*S*)-tetrahydrofuran-2-yl)methyl)naphthalene)-1-sulfonamido)phenyl)amino)-[1,1'-binaphthalen]-2-yl)amino)phenyl)-*N*-(((*S*)-tetrahydrofuran-2-yl)methyl)naphthalene-1-sulfonamide (14).

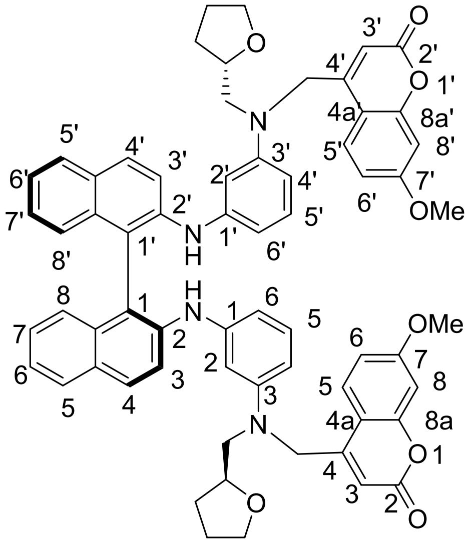

15

4,4'-((((((*S*)-[1,1'-binaphthalene]-2,2'-diyl)bis(azanediyl))bis(3,1-phenylene))bis((((*S*)-tetrahydrofuran-2-yl)methyl)azanediyl))bis(methylene))bis(7-methoxy-2H-chromen-2-one) (15).

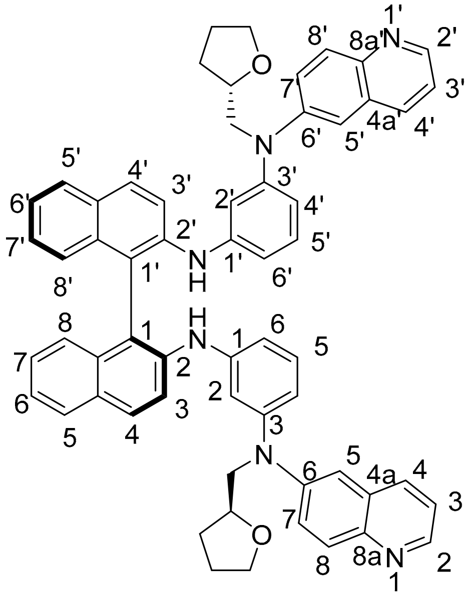

16

***N**^{1}**,*N**$^{1'}$**-((*S*)-[1,1'-binaphthalene]-2,2'-diyl)bis(*N*^{3}-(quinolin-6-yl)-*N*^{3}-(((*S*)-tetrahydrofuran-2-yl)methyl)benzene-1,3-diamine) (16).**

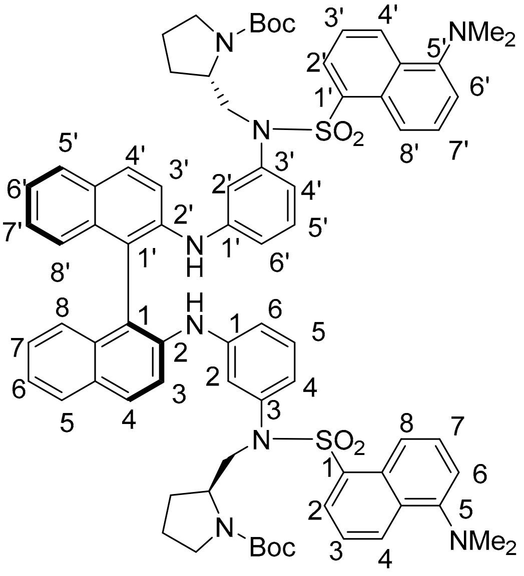

17

Di-*tert*-butyl-2,2'-((((((*S*)-[1,1'-binaphthalene]-2,2'-diyl)bis(azanediyl))bis(3,1-phenylene))bis(((5-(dimethylamino)naphthalen-1-yl)sulfonyl)azanediyl))bis(methylene))-(2*S*,2'*S*)-bis(pyrrolidine-1-carboxylate) (17).

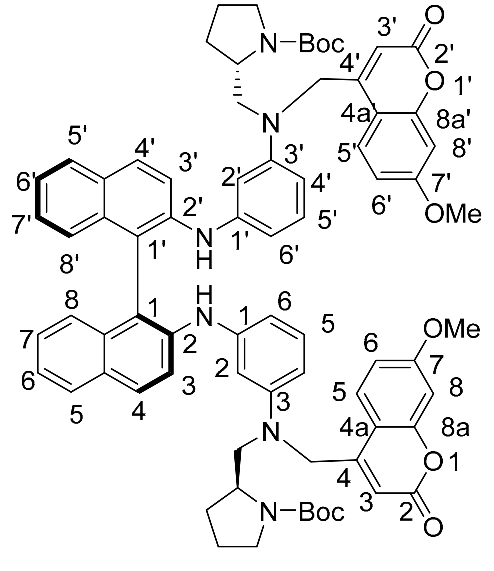

18

Di-*tert*-butyl-2,2'-((((((*S*)-[1,1'-binaphthalene]-2,2'-diyl)bis(azanediyl))bis(3,1-phenylene))bis(((7-methoxy-2-oxo-2H-chromen-4-yl)methyl)azanediyl))-bis(methylene))(2*S*,2'*S*)-bis(pyrrolidine-1-carboxylate) (18).

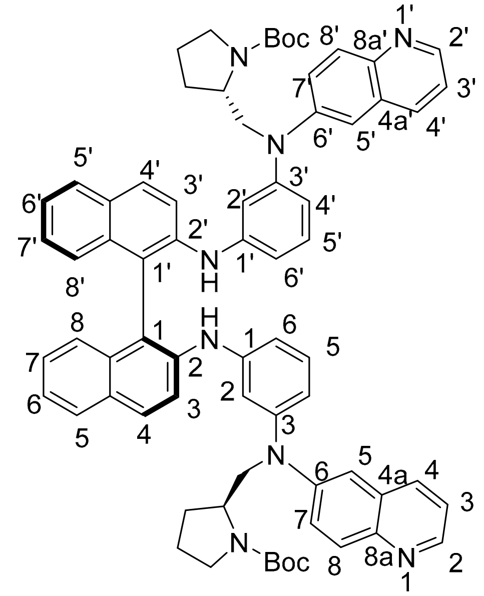

19

Di-*tert*-butyl-2,2'-((((((*S*)-[1,1'-binaphthalene]-2,2'-diyl)bis(azanediyl))bis(3,1-phenylene))bis(quinolin-6-ylazanediyl))bis(methylene))(2*S*,2'*S*)-bis(pyrrolidine-1-carboxylate) (19).

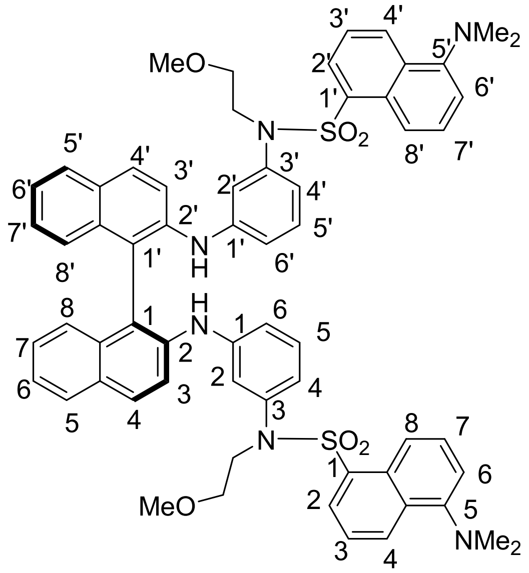

22

(*S*)-*N,N'*-(([1,1'-binaphthalene]-2,2'-diylbis(azanediyl))bis(3,1-phenylene))bis(5-(dimethylamino)-*N*-(2-methoxyethyl)naphthalene-1-sulfonamide) (22).

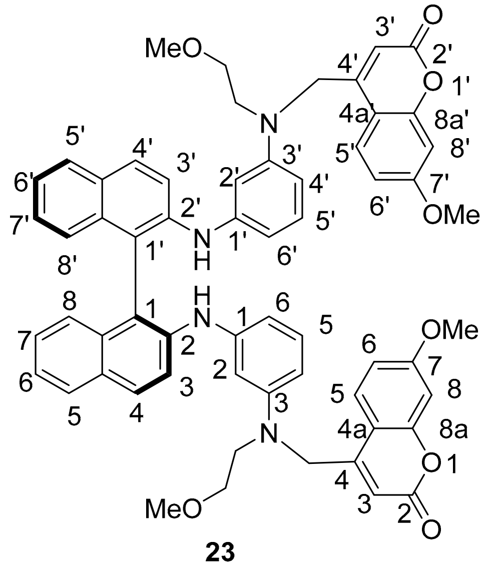

23

(*S*)-4,4'-((((([1,1'-binaphthalene]-2,2'-diylbis(azanediyl))bis(3,1-phenylene))bis((2-methoxyethyl)azanediyl))bis(methylene))bis(7-methoxy-2H-chromen-2-one) (23).

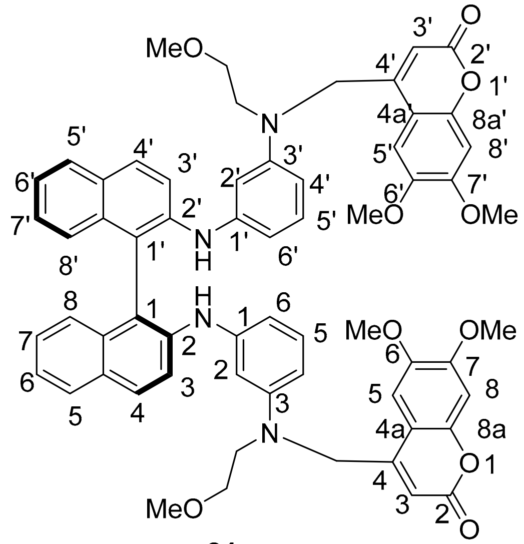

24

(*S*)-4,4'-((((([1,1'-binaphthalene]-2,2'-diylbis(azanediyl))bis(3,1-phenylene))bis((2-methoxyethyl)azanediyl))bis(methylene))bis(6,7-dimethoxy-2H-chromen-2-one) (24).

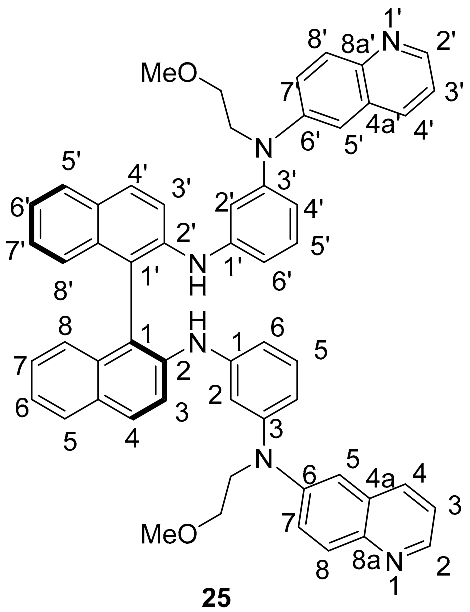

25

(*S*)-N^{1},$N^{1'}$-([1,1'-binaphthalene]-2,2'-diyl)bis(N^{3}-(2-methoxyethyl)-N^{3}-(quinolin-6-yl)benzene-1,3-diamine) (25).

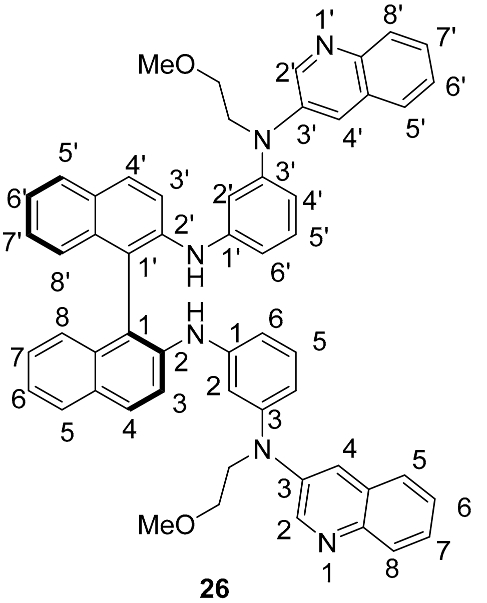

26

(*S*)-*N*[1],*N*[1′]-([1,1'-binaphthalene]-2,2'-diyl)bis(*N*[3]-(2-methoxyethyl)-*N*[3]-(quinolin-3-yl)benzene-1,3-diamine) (26).

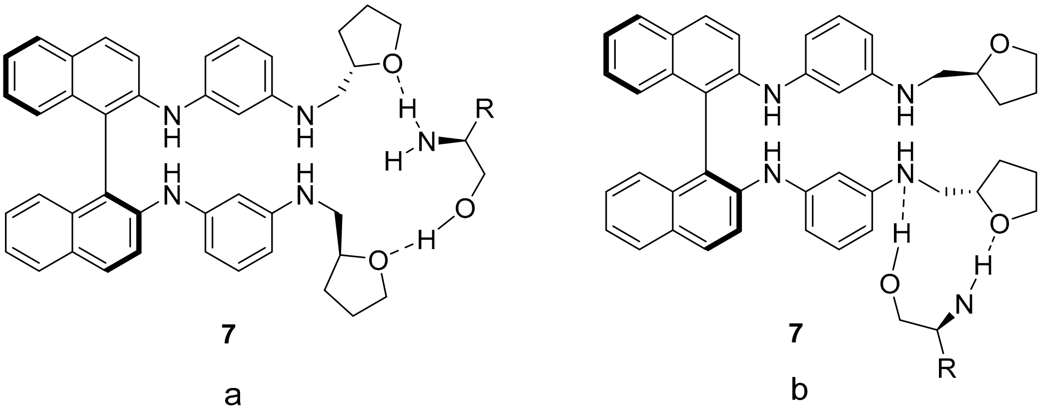

7 **7**

a b

Figure S1. Plausible coordination patterns for amino alcohols with the BINAM-based ligands: (**a**) coordination with two chiral suntsituents; (**b**) coordination with one chiral substituent.

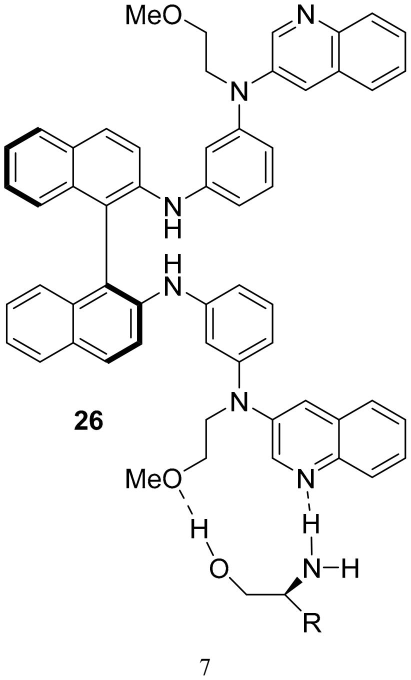

26

Figure S2. Plausible coordination of amino alcohols with the ligand **26**.

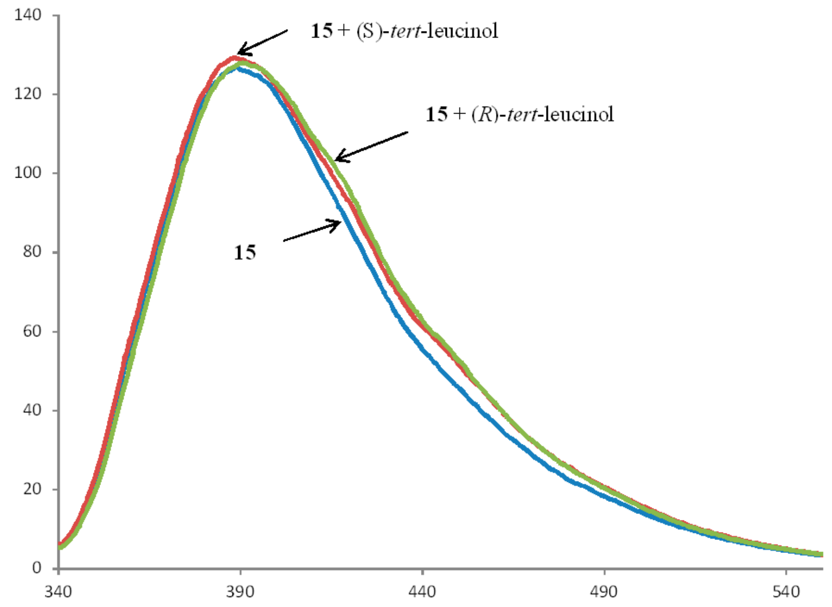

Figure S3. Fluorescence spectra of compound **15** in the presence of (*R*)- and (*S*)-enantiomers of *tert*-leucinol (1000 equiv.).

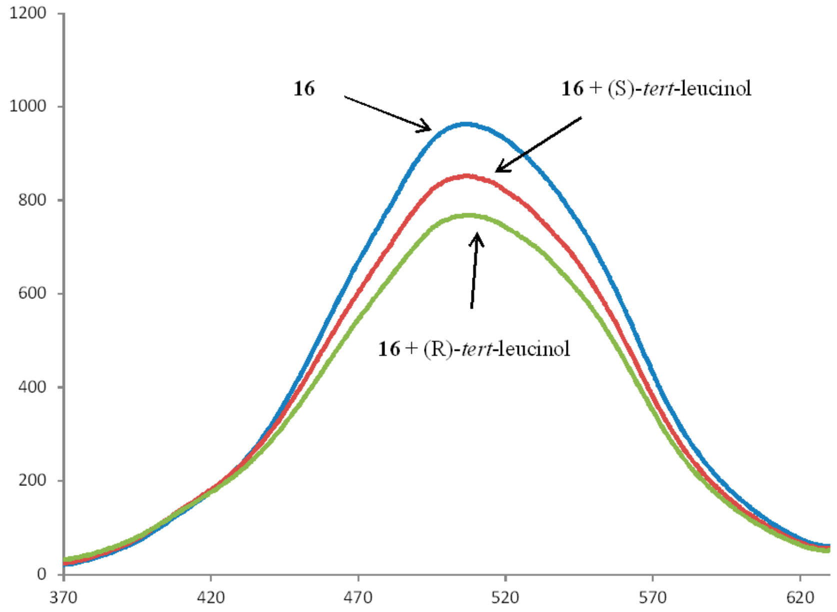

Figure S4. Fluorescence spectra of compound **16** in the presence of (*R*)- and (*S*)-enantiomers of *tert*-leucinol (1000 equiv.).

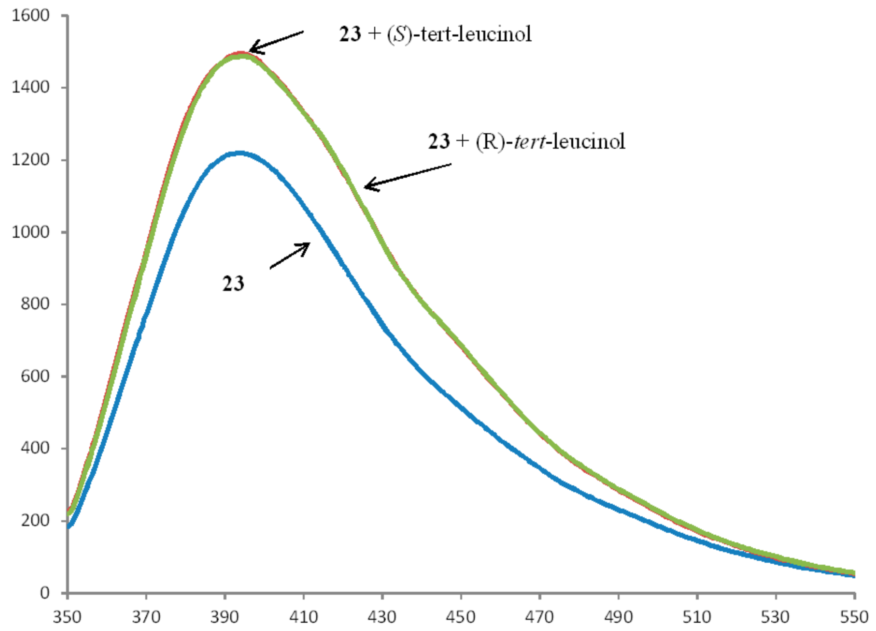

Figure S5. Fluorescence spectra of compound **23** in the presence of (*R*)- and (*S*)-enantiomers of *tert*-leucinol (1000 equiv.).

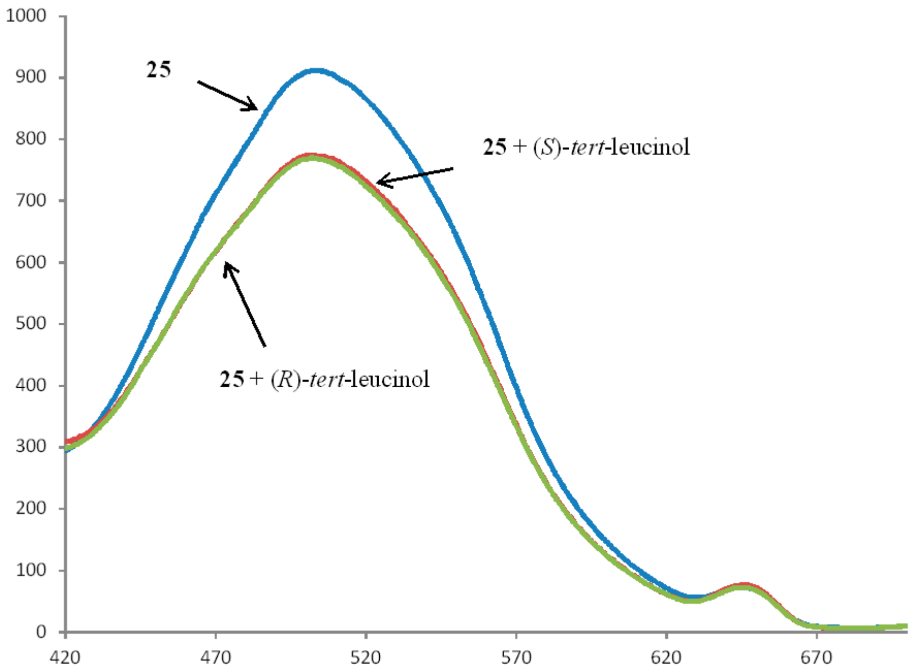

Figure S6. Fluorescence spectra of compound **25** in the presence of (*R*)- and (*S*)-enantiomers of *tert*-leucinol (1000 equiv.).

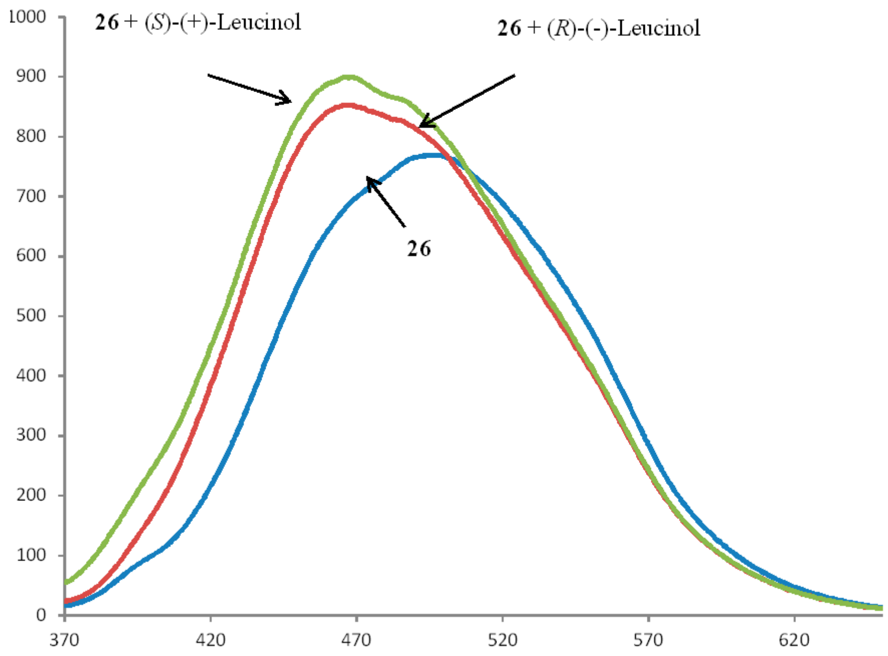

Figure S7. Fluorescence spectra of compound **26** in the presence of (*R*)- and (*S*)-enantiomers of leucinol (1000 equiv.).

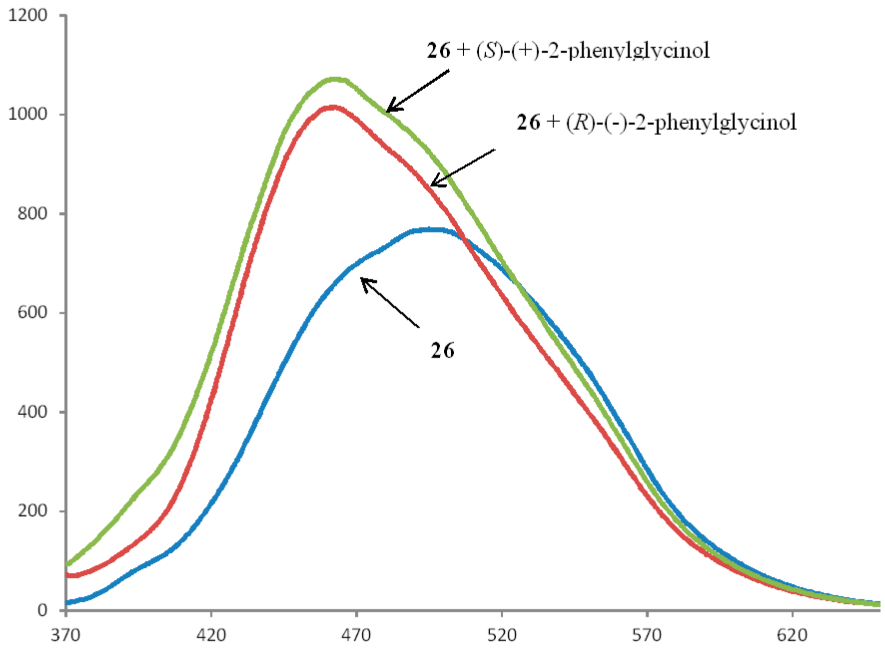

Figure S8. Fluorescence spectra of compound **26** in the presence of (*R*)- and (*S*)-enantiomers of 2-phenylglycinol (1000 equiv.).

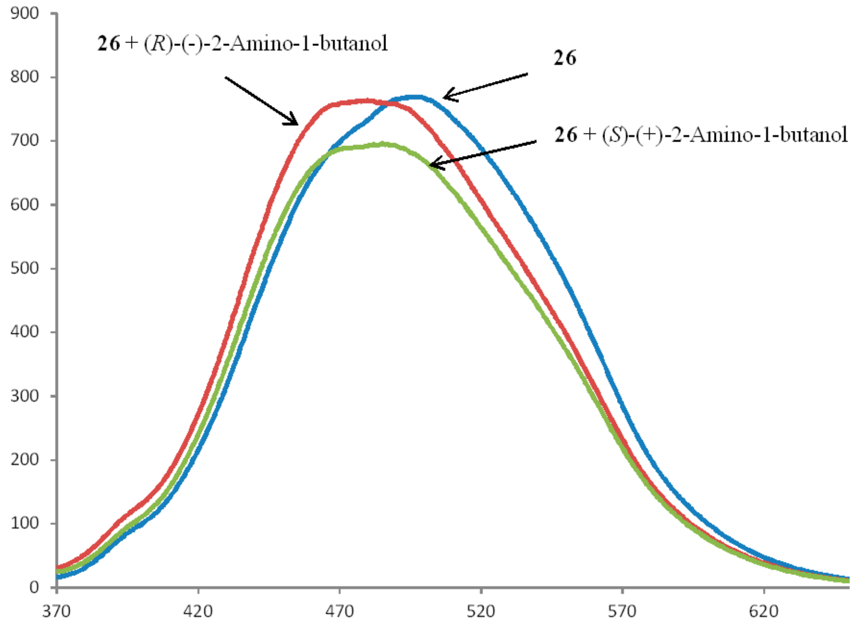

Figure S9. Fluorescence spectra of compound **26** in the presence of (*R*)- and (*S*)-enantiomers of 2-amino-1-butanol (1000 equiv.).

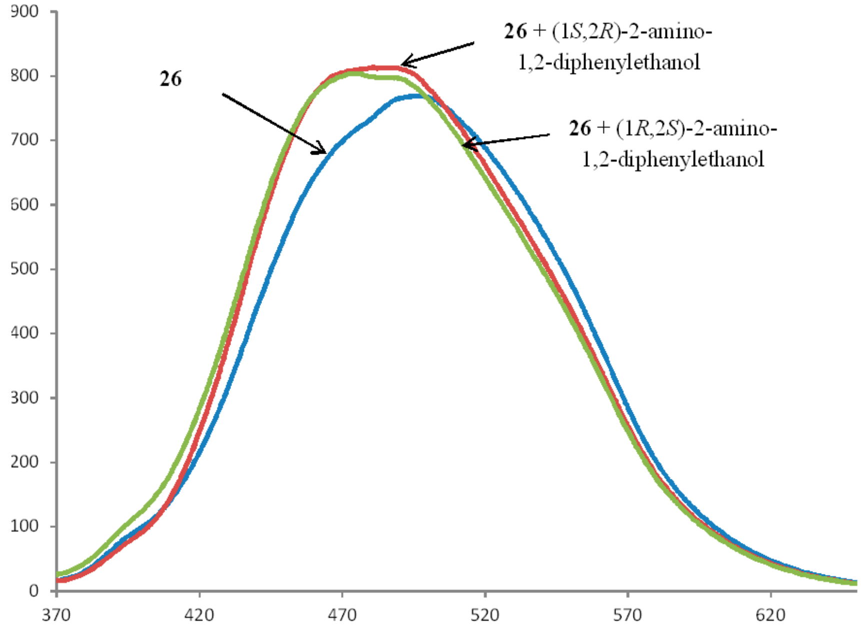

Figure S10. Fluorescence spectra of compound **26** in the presence of the enantiomers of 2-amino-1,2-diphenylethanol (1000 equiv.).
